# Single‐Cell RNA Sequencing Identifies Accumulation of Fcgr2b + Virtual Memory‐Like CD8 T Cells With Cytotoxic and Inflammatory Potential in Aged Mouse White Adipose Tissue

**DOI:** 10.1111/acel.70278

**Published:** 2025-10-21

**Authors:** Archit Kumar, Martin O'Brien, Vincent B. Young, Raymond Yung

**Affiliations:** ^1^ Department of Internal Medicine, Division of Geriatric and Palliative Medicine University of Michigan Medical School Ann Arbor Michigan USA; ^2^ Department of Internal Medicine, Division of Infectious Diseases, AND Department of Microbiology and Immunology University of Michigan Medical School Ann Arbor Michigan USA

**Keywords:** adipose tissue, aging, CD8 T cells, memory T cells, obesity, single‐cell analysis

## Abstract

Aging and obesity are associated with pro‐inflammatory changes in adipose tissue. Overlapping mechanisms, such as the infiltration of inflammatory macrophages and T cells into visceral adipose tissue, have been implicated in contributing to inflammation. However, a comparative analysis of both states is needed to identify distinct regulatory targets. Here, we performed single‐cell RNA sequencing of stromal vascular fractions (SVF) isolated from gonadal white adipose tissue (gWAT) of young mice fed either a normal or a high‐fat diet, and aged mice fed a normal diet. Our analysis revealed that physiological aging, compared to high‐fat diet‐induced obesity, was associated with an accumulation of phenotypically distinct CD8 T cells resembling virtual memory (VM) CD8 T cells. These cells expressed high levels of *Cd44*, *Sell*, *Il7r*, *Il2rb*, lacked *Itga4*, and exhibited elevated *Fcgr2b* expression which was associated with pseudotime differentiation trajectories. Flow cytometry confirmed an age‐associated increase in Fcgr2b + CD49d‐ VM‐like CD8 T cells in gWAT. Notably, these Fcgr2b‐expressing cells exhibited a cytotoxic profile and expressed granzyme M. Functional analysis using recombinant granzyme M revealed its potential in inducing inflammation in mouse fibroblasts and macrophages. Together, our study has identified Fcgr2b + CD49d‐ VM‐like CD8 T cells in the adipose tissue of aged mice with regulatory, cytotoxic, and inflammatory potential.

## Graphical abstract

Age‐associated accumulation of Fcgr2b + VM‐like CD8 T cells within visceral adipose tissue contributes to inflammation.

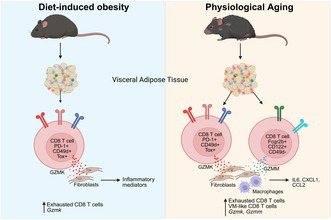

## 1 Introduction

Aging is an inevitable biological process characterized by the progressive deterioration of physiological functions, which can lead to increased vulnerability to disease and death. During aging, host immune cells lose their ability to respond to infections and cancer, while simultaneously acquiring a pro‐inflammatory phenotype that contributes to tissue pathology. Moreover, chronic low‐grade inflammation associated with aging, termed “inflammaging”, is a major driver of age‐related diseases including type 2 diabetes, metabolic syndrome, cardiovascular diseases, and cancer (Leonardi et al. 2018; Franceschi et al. 2018; Ferrucci and Fabbri 2018). Many of these age‐related diseases are also seen in obesity, suggesting that obesity can accelerate the aging process (Santos and Sinha 2021). Adipose tissue is one of the notable organs affected during aging, where age‐related changes in immune cells are first detected (Schaum et al. 2020). Moreover, an increase in body weight, accumulation of visceral fat mass, and adipose tissue dysfunction are associated with both aging and obesity (Ou et al. 2022; Reyes‐Farias et al. 2021; Trim et al. 2018).

Adipose tissue (AT) is a metabolically active endocrine organ distributed throughout the body, playing a central role in regulating systemic metabolism through the secretion of adipokines and cytokines. AT comprises mainly brown adipose tissue (BAT) and white adipose tissue (WAT). BAT, predominantly located in the supraclavicular and interscapular regions (Sacks and Symonds 2013), is essential for thermogenesis, while WAT functions primarily as an energy reservoir. The metabolic activity and plasticity of WAT are highly responsive to changes in energy supply and demand. WAT can be further subdivided into visceral adipose tissue (VAT), which surrounds internal organs, and subcutaneous adipose tissue (SCAT), which is located beneath the skin. Accumulation of VAT is strongly associated with increased risk of cardiovascular diseases, metabolic disorders, and cancer (Medina‐Urrutia et al. 2025; Zhang et al. 2025; Nguyen and Shanmugan 2024; Vasamsetti et al. 2023). Structurally, WAT is a complex and heterogeneous tissue composed of adipocytes, mesenchymal stromal cells, endothelial cells, and immune cells. Although adipocytes dominate the tissue structural volume of WAT, a substantial portion of its cellular composition consists of nonadipocyte cells, collectively known as stromal vascular fraction (SVF). Functional changes in SVF of adipose tissue, especially fibroblasts and immune cells, play a critical role in regulating inflammation and metabolic homeostasis (Ou et al. 2022; Reyes‐Farias et al. 2021; Trim et al. 2018).

Both aging and diet‐induced obesity significantly alter the heterogeneity and function of adipose tissue. These changes include secretion of senescent‐associated secretory phenotype (SASP) proteins, acquisition of pro‐inflammatory phenotype, accumulation of T cells, γδ T cells, B cells, and inflammatory macrophages in VAT (Camell et al. 2019; Lumeng et al. 2011; Winer et al. 2011; Nishimura et al. 2009; Weisberg et al. 2003; Bruno et al. 2022; Mukherjee et al. 2023). Recent advancements such as single‐cell (sc) and single‐nuclei (sn) RNA sequencing (RNAseq) have been employed to investigate these alterations in the VAT, especially in gonadal white adipose tissue (gWAT), during aging and diet‐induced obesity in mice (Wang et al. 2025; So et al. 2025; Wu et al. 2024; Liao et al. 2024; Kar et al. 2024; Muñoz‐Rojas et al. 2024; Emont et al. 2023; Cottam et al. 2022; Sárvári et al. 2021; Mogilenko et al. 2021). Although recent studies have revealed important insights into the regulation of fibroblasts, regulatory T cells (Tregs), and exhausted T cells in adipose tissue, a comparative analysis of aging‐ and obesity‐associated changes remains lacking. To address this gap, we conducted an integrated scRNAseq analysis of SVF isolated from gWAT of aged and obese mice of both sexes. Our findings revealed distinct differences in CD8 T cell memory subsets between aged and obese gWAT in mice. Specifically, memory CD8 T cells in aged mice express high levels of inhibitory receptor *Fcgr2b*, exhibit a cytotoxic profile, and could initiate granzyme M‐mediated inflammatory responses in mouse fibroblasts and macrophages.

## 2 Results

### 2.1 Aging and High‐Fat Diet Induces gWAT Dysfunction in Mice

**FIGURE 1:**
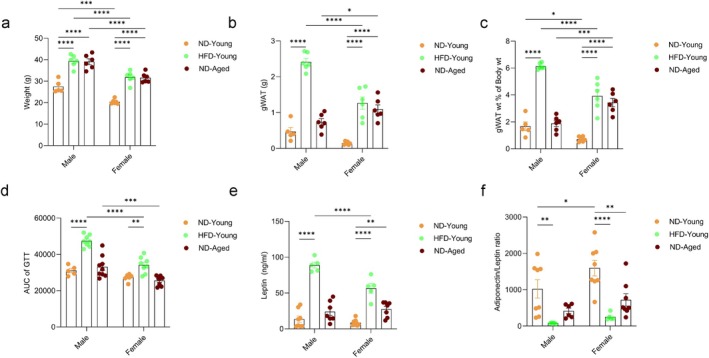
Aging and HFD‐induced obesity impair gWAT function. (a) Body weight, (b) gWAT mass, (c) gWAT mass normalized to body weight in normal diet (ND)‐fed young mice (4–5 months old), high‐fat diet (HFD)‐fed young mice (42% Kcal from fat for 12 weeks, 4–5 months old), and ND‐fed aged mice (21–24 months old) of both sexes. (d) Area under the curve (AUC) of serum glucose levels during a glucose tolerance test (GTT) following overnight fasting. (e) Serum leptin levels. (f) Serum adiponectin‐to‐leptin ratio. Data are presented as mean ± SEM. Statistical significance was determined using two‐way ANOVA followed by Tukey's HSD post hoc test. *N* = 5–8 mice per group. *p* values < 0.05 were considered significant. *p < 0.05, **p < 0.01, ***p < 0.001, ****p < 0.0001.

Visceral adiposity induced during high‐fat diet (HFD) exposure or aging can influence the development of metabolic syndrome and systemic inflammation. To investigate the similarities and differences in adipose tissue dysfunction under these conditions, we assessed metabolic stress responses and circulating adipokine levels in young mice (4–5 months old) fed a normal diet (ND) or HFD (42% Kcal from fat) for 12 weeks and aged mice (21–24 months old) maintained on ND. While both HFD exposure in young mice and physiological aging in ND‐fed mice significantly increased body weight, a significant increase in gonadal white adipose (gWAT) mass was observed in HFD‐fed mice (male and female) and in ND‐fed aged female mice (Figure 1a–c). Male mice generally exhibited significantly higher body weight compared to female mice. Notably, HFD‐fed young male mice displayed greater adiposity than their female counterparts, while aged female mice exhibited more pronounced fat accumulation (gWAT mass) than aged males. In response to metabolic stress, only HFD‐fed young mice exhibited metabolic syndrome when compared to ND‐fed young mice, as indicated by significantly elevated blood glucose levels during the glucose tolerance test (GTT) and under fasting (Figures 1d and S1a–c). HFD‐fed young male mice also showed increased serum glucose levels during the insulin tolerance test (ITT), although the differences were not statistically significant when compared to ND‐fed young male mice (Figure S1c,d,f). Furthermore, male mice exhibited significantly worse outcomes during metabolic stress tests under both aging and HFD‐induced obesity conditions, compared to females of the same cohorts.

Dysregulation of adipokine levels, primarily adiponectin and leptin, are well established indicators of impaired adipose tissue function. To assess these changes under different physiological conditions, we measured serum adipokine levels in young mice subjected to ND or HFD, and in aged mice maintained on ND. HFD‐fed young mice exhibited significantly elevated serum leptin levels and reduced adiponectin‐to‐leptin ratios relative to ND‐fed young mice (Figure 1e,f). Conversely, age‐associated changes in the leptin levels were more pronounced in female mice compared to ND‐fed young female mice. Female mice across all the experimental groups showed elevated adiponectin levels; however, no significant sex‐specific differences in the adiponectin‐to‐leptin ratio were observed under either HFD or aging conditions, although only ND‐fed young females exhibited a significantly higher adiponectin‐to‐leptin ratio compared to ND‐fed young mice (Figures 1e,f and S1g). The results suggest sex‐specific regulation of adipokines and adiposity where female mice may exert a more protective role against metabolic dysfunction than male mice. However, this protection appears to be diminished with aging and HFD‐induced obesity.

### 2.2 Aged Mice Exhibit Altered Adipose Tissue Cellular Heterogeneity

**FIGURE 2:**
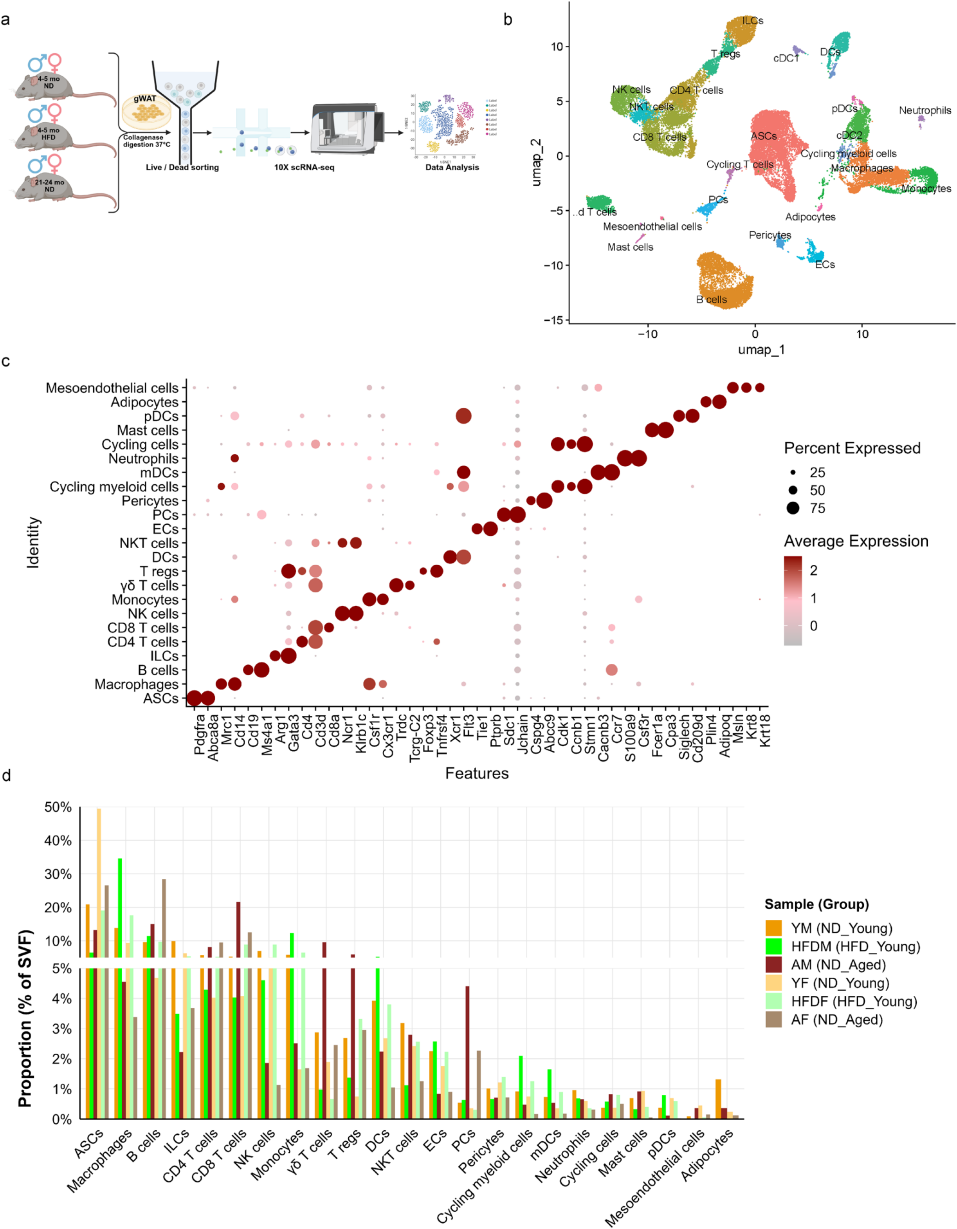
scRNA‐seq analysis of stromal vascular fraction of gWAT. (a) Schematic overview of single‐cell RNA sequencing (scRNA‐seq) performed on SVF isolated from gWAT of ND‐fed young, HFD‐fed young, and ND‐fed aged mice (both sexes), created using BioRender.com. Each scRNA‐seq sample was generated by pooling SVF from three independent mice per group per sex after live/dead cell sorting. (b) UMAP plot showing 32,806 cells after quality control and data integration, colored by annotated cell types. (c) Dot plot showing expression of marker genes across identified cell‐types. (d) Bar plot representing the proportions of nonimmune and immune cell types in the SVF. ASCs, Adipose stem‐like cells; DCs, Dendritic cells; ECs, Endothelial cells; ILCs, Innate lymphoid cells; mDCs, Migratory dendritic cells; NK, Natural killer cells; NKT, Natural killer T cells; PCs, Plasma cells; pDCs, Plasmacytoid dendritic cells; T regs, T regulatory cells; γδ T cells, Gamma delta T cells.

To characterize differences in adipose tissue heterogeneity during HFD exposure and aging, we performed scRNA‐seq on SVF isolated from gWAT of ND‐fed young, HFD‐fed young, and ND‐fed aged mice of both sexes. SVF from each individual mouse (*n* = 3 per group per sex, 18 mice total) was sorted to remove dead cells, and an equal number of viable cells from each mouse within a group were pooled to generate three distinct experimental groups per sex (yielding six pooled samples total) (Figure 2a). Libraries were prepared using the 10× Genomics Chromium Single Cell 3′ platform and subjected to Illumina sequencing. After quality control and filtering, 32,806 cells were retained and integrated using Seurat. Dimensionality reduction with Uniform Manifold Approximation and Projection (UMAP) was used to visualize the data (Figure 2b). Cell type annotation was performed based on the expression of canonical and previously established marker genes (Figure 2c and S2) (Liao et al. 2024; Muñoz‐Rojas et al. 2024). Both HFD‐fed young mice and ND‐fed aged mice exhibited a reduction in adipose stem‐like cell (ASCs) populations, particularly in males across all groups (Figure 2d). ASCs heterogeneity has been shown to vary due to diet, age, sex, and plays a significant role in regulating adiposity (Wu et al. 2024; Liao et al. 2024; Kar et al. 2024; Sárvári et al. 2021). In line with previous literature, HFD‐fed mice showed increased infiltration of macrophages, whereas ND‐fed aged mice demonstrated greater accumulation of B cells, CD8 T cells, γδ T cells, regulatory T cells (Tregs), and PCs in gWAT (Camell 2022; Camell et al. 2019; Bénézech et al. 2015; Lumeng et al. 2011; Bruno et al. 2022; Mukherjee et al. 2023; Bapat et al. 2015; Kohlgruber et al. 2018) (Figure 2d). The accumulation of CD8 T cells, γδ T cells, and Tregs was more pronounced in ND‐fed aged male mice, while B cell accumulation was higher in ND‐fed aged female mice (Figure 2d). These findings highlight distinct heterogeneity in the immune cells of adipose tissue during aging and diet‐induced obesity.

### 2.3 CD8 T Cells in Aged Mice Exhibit Enhanced Cell–Cell Communication

**FIGURE 3:**
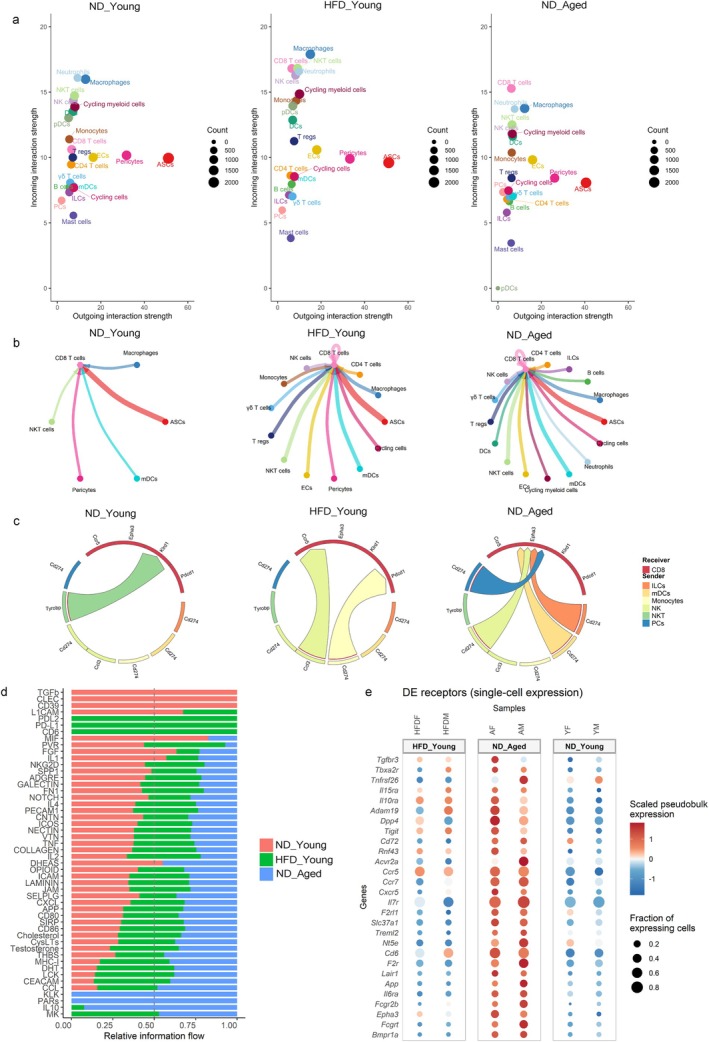
Cell–cell interaction within SVF. (a) Dot plots representing incoming and outgoing signaling strengths between cell types within the SVF of ND‐fed young, HFD‐fed young, and ND‐fed aged mice inferred using CellChat. Dot size reflects the number of cells within each cell type. (b) Circle plot showing incoming signals to CD8 T cells from other cell types based on ligand‐receptor (L‐R) expression. Each node represents a cell type, and edge width indicates interaction strength. (c) Chord diagram representing incoming signals to CD8 T cells inferred using MultiNicheNet. The top 100 differentially expressed L‐R pairs among ND‐fed young, HFD‐fed young, and ND‐fed aged mice were used to generate the plots. Edge width represents the predicted strength of incoming signals to CD8 T cells. (d) Bar graphs showing the strength of signaling pathways targeting CD8 T cells in each group, generated using RankNet() function in CellChat. (e) Dot plot displaying receptor genes upregulated in CD8 T cells (logFC > 0.5 and *p* values ≤ 0.05). Dot size represents the percentage of CD8 T cells expressing each receptor gene, and color indicates the average expression level.

Following the identification of altered cellular heterogeneity in gWAT during aging and obesity, we next investigated cell–cell communication to identify interacting cell types. We employed CellChat, a computational tool that infers intercellular signaling networks based on curated ligand‐receptor (L‐R) interactions, to quantify signaling dynamics in young mice either fed ND or HFD, and in ND‐fed aged mice (Jin et al. 2025). CellChat analysis was performed both on aggregated male and female samples of each group (Figure 3a) and on individual samples (Figure S3). Our analysis revealed that CD8 T cells from both HFD‐fed young mice and ND‐fed aged mice displayed increased incoming signaling strength, indicating a higher degree of interaction with other gWAT cell types compared to CD8 T cells from ND‐fed young mice (Figure 3a). However, no sex‐related changes were observed in the signaling strengths of cell–cell communication (Figure S3). Examination of incoming signals indicated a broad engagement of CD8 T cells with various cell populations under both aging and HFD conditions (Figure 3b).

While CellChat provides a global view of intercellular communication, it does not resolve differences arising from differential expression of L‐R pairs between groups. Therefore, we applied MultiNicheNet to identify age and diet specific communication based on differentially expressed L‐R gene pairs (Browaeys et al. 2023). As MultiNicheNet requires at least two samples per group to increase statistical power, this analysis was performed on aggregated male and female samples within each group. This analysis revealed that, during aging, most SVF cells uniquely signaled to CD8 T cells, primarily through MHC class Ib molecules and *Cd274* (Figures 3c and S4). In addition to MHC‐1b and Cd274 mediated signaling, we also observed cell‐type specific interactions: Tregs, γδT cells and endothelial cells (ECs) signaled to CD8 T cells via *Il10*, *Bmp4/6*, and *Inhbb*, while ILCs, NK cells and neutrophils signaled through *Tgfb1*.

To evaluate global shifts in signaling pathways, we applied the RankNet function of CellChat, which showed age‐related enrichment in IL10, PARs (protease‐activated receptors), KLK (kallikrein‐related peptidases), CCL (chemokines), CEACAM (adhesion molecules), and MHC‐I signaling pathways (Figure 3d). Enrichment in MHC‐I and IL10 signaling pathways in aged mice suggests the development of cytotoxic and exhausted phenotype of CD8 T cells (Smith et al. 2018; Raskov et al. 2021). Further analysis of receptor expression in aged CD8 T cells using MultiNicheNet revealed increased transcription of *Il15ra*, *Il10ra*, *Il7r*, *Ccr7*, *Acvr2a*, *Fcgr2b*, and *Epha3* (Figure 3e and S5). Notably, *Il7r* and *Ccr7* are markers of naïve and central memory (CM) CD8 T cells, facilitating their homing to lymphoid structures. Given the well‐established age‐associated decline in naïve CD8 T cells (Goronzy et al. 2015), we hypothesize that increased expression of *Il7r* and *Ccr7* reflects the accumulation of CM CD8 T cells in aged gWAT. Overall, our cell–cell communication analysis suggests that the aged gWAT microenvironment modulates CD8 T cell phenotype towards an immunosuppressive phenotype, through signaling mediated by *Cd274*, MHC‐1b, *Il10*, *Bmp4/6*, and *Tgfb2*.

### 2.4 CD8 T Cells in Aged Mice Exhibit a Distinct Phenotypic Landscape

**FIGURE 4:**
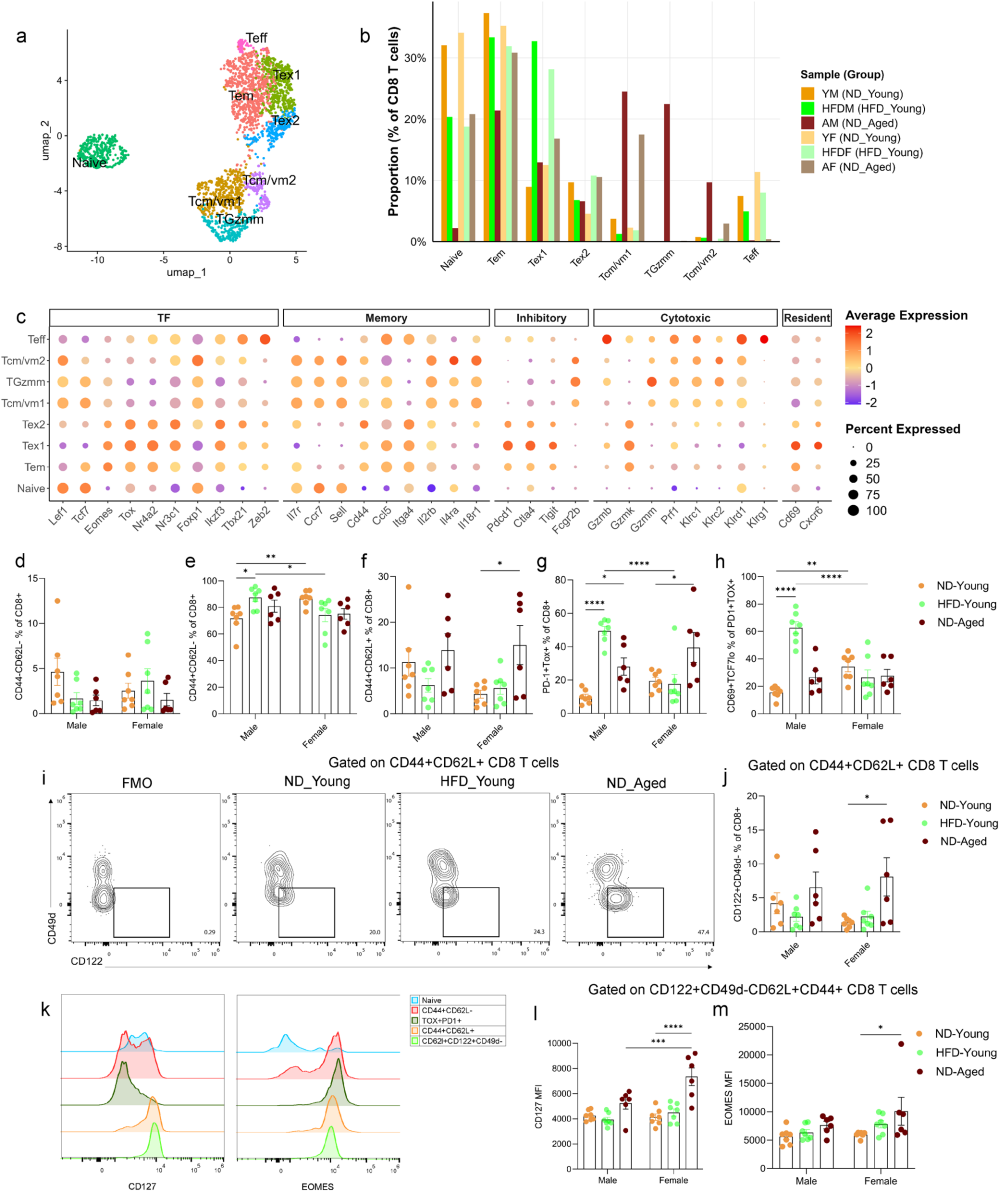
scRNA‐seq analysis of CD8 T cells. (a) UMAP plot showing 2408 CD8 T cells following subsetting from SVF of ND‐fed young, HFD‐fed young, and ND‐fed aged mice, colored by annotated cell types. (b) Bar plot representing the proportions of CD8 T cell subsets relative to total CD8 T cells. (c) Dot plot displaying the expression of marker genes used to define CD8 T cell subsets. Dot size represents the fraction of cells expressing each marker gene and color indicates the average expression across subsets. Bar plots showing frequencies of (d) CD44‐CD62L‐, (e) CD44 + CD62L‐, (f) CD44 + CD62L+, and (g) PD‐1 + Tox + within CD8 T cells measured using flow cytometry across different groups. (h) Bar plot showing frequencies of CD69 + TCF7lo cells within PD‐1 + Tox + CD8 T cells. (i) Contour plot representing gating strategy, (j) Bar plot showing the frequency of CD122 + CD49d‐ virtual memory CD8 T cells across different groups. (k) Histograms illustrating the expression of CD127(*Il7r*) and EOMES in various CD8 T cell subsets. Bar graphs representing the median fluorescent intensity of (l) CD127 and (m) EOMES on CD122 + CD49d‐CD62L + CD44+ CD8 T cells across different groups. Data are presented as mean ± SEM. Statistical significance was determined using two‐way ANOVA followed by Tukey's HSD post hoc test. Only significant comparisons are shown between the sex of each group and with respect to ND‐fed young mice. *N* = 6–7 mice per group. *p* values < 0.05 were considered significant. *p < 0.05, **p < 0.01, ***p < 0.001, ****p < 0.0001. The gating strategy used to plot the cell types is shown in Figure S8.

To further characterize the phenotypic diversity of CD8 T cells in gWAT, we subsetted and reclustered the CD8a‐expressing population from SVF (shown in Figure 2b). Dimensionality reduction and reanalysis of 2408 CD8 T cells using UMAP revealed eight distinct clusters (Figure 4a). Cluster identities were assigned based on the expression of canonical marker genes (Figures 4c and S6). Naïve CD8 T cells were identified by high expression of stemness‐ and survival‐associated genes, including *Lef1*, *Tcf7*, *Foxp1*, and *Il7r*, along with *Ccr7* and *Sell*. Effector memory (EM) T cells (Tem) were distinguished by high expression of memory‐associated genes (*Eomes*, *Cd44*, *Ccl5*) and intermediate expression of exhaustion markers (*Pdcd1*, *Ctla4*). Two clusters (Tex1 and Tex2) represented exhausted CD8 T cells, characterized by elevated expression of *Tox*, *Nr4a2*, *Nr3c1*, *Ikzf3*, *Pdcd1*, and *Ctla4* and reduced expression of *Tcf7*. Tex1 also expressed high levels of the tissue‐residency markers *Cd69* and *Cxcr6*, indicating a residency phenotype acquired by these exhausted CD8 T cells. Both effector memory and exhausted CD8 T cells expressed high levels of *Gzmk*, which has previously been associated with inflammaging (Mogilenko et al. 2021). Transcript levels of *Gzmk* were high in both aged and obese mice (Figure S5). Clusters Tcm/vm1, Tcm/vm2, and TGzmm displayed features consistent with classical or virtual memory‐like CD8 T cells. These clusters showed elevated expression of central memory (CM) markers (*Cd44*, *Ccr7*, *Sell*) and lacked *Itga4* (Cd49d), an integrin upregulated upon antigen exposure, supporting a virtual memory (VM)‐like identity (Hussain and Quinn 2019; Chiu et al. 2013; Clambey et al. 2008). VM CD8 T cells, which arise independently of antigen stimulation, rely on cytokines such as IL‐7, IL‐15, and IL‐18 for maintenance, and accordingly they expressed *Il2rb*, *Il4ra*, *Il7r*, and *Il18r1* (Hussain and Quinn 2019). Cluster TGzmm, observed in ND‐fed male mice, further expressed *Gzmm* and *Prf1*, suggesting a cytotoxic function of these VM‐like CD8 T cells (Voskoboinik et al. 2015). Moreover, CM/VM‐like CD8 T cells exhibited high expression of regulatory/inhibitory receptors including *Fcgr2b*, *Acvr2a*, *Klrc1* and *Klrd1* (Figures 4c and S6). The Teff cluster was annotated as effector CD8 T cells based on high expression of cytotoxicity‐related genes *Tbx21*, *Zeb2*, *Gzmb*, *Prf1*, *Klrd1*, and *Klrg1*. As expected, HFD‐fed mice and ND‐fed aged mice displayed elevated frequencies of exhausted CD8 T cells (Tex1). However, age‐related changes were more pronounced in Tcm/vm1, Tcm/vm2, and TGzmm clusters compared to either ND‐ or HFD‐fed young mice (Figure 4b).

To validate the scRNA‐seq findings, we performed multicolor flow cytometry on SVF isolated from gWAT in a separate cohort of mice. Although naïve (CD44‐CD62L‐) CD8+ T cell infiltration was reduced in gWAT of ND‐fed aged mice compared to ND‐fed young mice, this difference was not statistically significant (Figure 4d). As expected, HFD‐fed male mice exhibited significant accumulation of PD‐1+ TOX+ and PD‐1+ TOX+ CD69+ TCF‐7lo CD8+ T cells, aligning with the exhausted phenotype seen in scRNA‐seq data (Figure 4g,h). HFD‐fed males also showed a significant increase in EM (CD44 + CD62L‐) CD8+ T cells. Since exhausted CD8+ T cells also express a CD44 + CD62L‐ phenotype (Figure 4c), increased frequencies of PD‐1+ cells on CD8+ T cells in flow cytometry may lead to overestimation of the EM population. In aged mice, we observed a significant accumulation of PD‐1+ TOX+ CD8+ T cells. However, the frequency of PD‐1+ TOX+ CD69+ TCF‐7lo CD8+ T cells was elevated in aged males, without reaching significance, suggesting that HFD exerts a more robust effect on the transition to exhaustion than physiological aging. Notably, aging was associated with significant increases in CM (CD44 + CD62L+) and VM‐like (CD44 + CD62L + CD122 + CD49d‐) CD8+ T cell subsets (Figure 4f,i,j), consistent with scRNA‐seq results (Figure 4b). These VM‐like CD8+ T cells expressed higher levels of CD127 (*Il7r*) and maintained Eomes expression (Figure 4k). Compared with young controls, aged VM‐like CD8+ T cells exhibited significantly elevated levels of CD127, while EOMES levels largely remained unchanged (Figure 4l,m). Although these protein expression changes were more pronounced in aged female mice, expansion of VM‐like CD8+ T cells during aging suggests a unique and potentially critical role for these cells in adipose tissue during aging.

### 2.5 Trajectory Analysis Reveals Dysregulated Differentiation Stages of Aged CD8 T Cells

**FIGURE 5:**
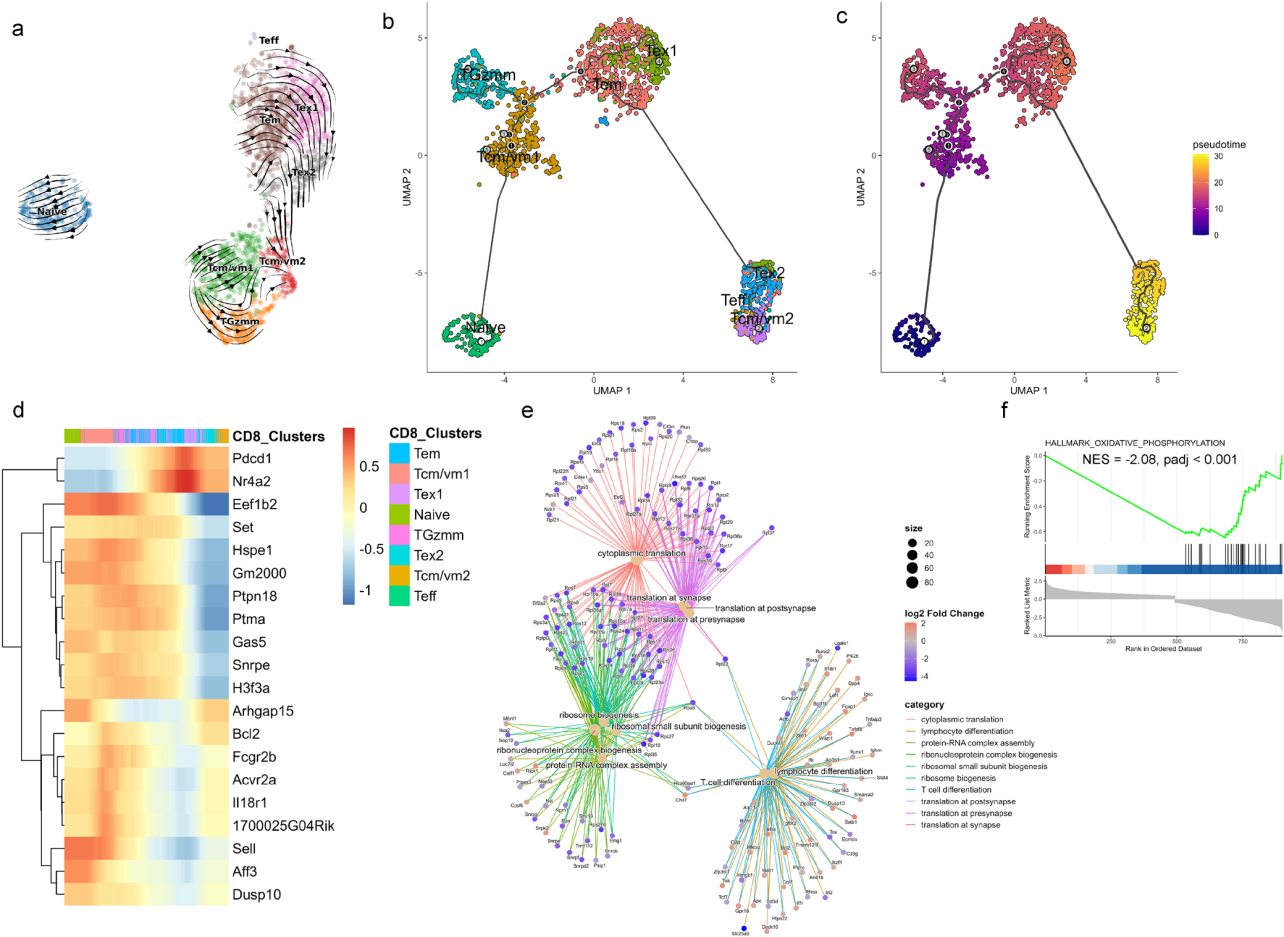
RNA velocity and trajectory analysis of aged CD8 T cells. (a) UMAP plot showing trajectories inferred from RNA velocity, (b) trajectory constructed using Monocle, and (c) pseudotime projection of CD8 T cells from ND‐fed aged mice. (d) Heatmap showing the dynamic changes in gene expression along pseudotime, (e) cnetplot illustrating functional relationships among the top 10 significant Gene Ontology (GO) terms and their associated genes in the Tcm/vm^2^ cluster. Node size reflects *p* value of each GO term, and color indicates the log2 fold change in gene expression. (f) Gene set enrichment analysis (GSEA) enrichment plot for Hallmark oxidative phosphorylation gene set in the Tcm/vm2 cluster.

To explore the differentiation dynamics of CD8 T cells in aged gWAT, we performed RNA velocity analysis, Monocle trajectory inference, and pseudotime analysis on ND‐fed aged mice (aggregated male and female). RNA velocity estimates future cell states based on spliced and unspliced mRNA ratios, while Monocle infers differentiation trajectories based on gene expression changes along pseudotime (Trapnell et al. 2014; Bergen et al. 2020; La Manno et al. 2018). Both methods revealed similar trends in the developmental trajectories (Figure 5a–c). Monocle inferred pseudotime trajectories suggested a progression from the Tcm/vm1 cluster towards the Tem cluster, consistent with prior reports of homeostatic, antigen‐independent proliferation from CM to EM cells (Bouneaud et al. 2005; Geginat et al. 2003, 2001). Both RNA velocity and Monocle predicted that memory subsets could subsequently transition into a Tcm/vm2 phenotype. Tem cells could differentiate into exhausted subsets (Tex1 and Tex2), which could further transition into a Tcm/vm2 phenotype. Additionally, cluster TGzmm appeared to be differentiated from Tcm/vm1, and both TGzmm and Tcm/vm1 cells at later stages could converge towards the Tcm/vm2 phenotype.

To further define these differentiation pathways, we plotted the top 20 differentially expressed genes along pseudotime in a heatmap (Figure 5d). Cells at the earliest pseudotime points exhibited high expression of *Sell* and *Dusp10*, indicative of a naïve phenotype. The transition towards a CM/VM‐like phenotype was marked by increased expression of *Fcgr2b*, *Acvr2a*, and *Il18r1*, alongside sustained *Sell* expression. Differentiation of TGzmm cells from Tcm/vm1 was characterized by high expression of *Fcgr2b* and intermediate levels of *Sell* and *Il18r1* (Figures 4c and 5d). Acquisition of an exhaustion phenotype (Tex1) was associated with upregulation of *Pdcd1* and *Nr4a2*. Differentiation into the Tcm/vm2 subset was marked by downregulation of genes involved in protein synthesis (*Eef1b2*, *Hspe1*, *Snrpe*, *H3f3a*), by intermediate expression of *Fcgr2b*, *Dusp10*, and *Il18r1*, and by high expression of *Bcl2*. This transcriptional profile suggests that Tcm/vm2 cells possess reduced translational activity while maintaining survival potential and immunological responsiveness. To functionally characterize the Tcm/vm2 population, we performed overrepresentation analysis and Gene Set Enrichment Analysis (GSEA) which revealed significant defects in protein translation, ribosome biogenesis, and oxidative phosphorylation pathways (Figure 5e,f). At the same time, expression of *Lef1*, *Foxp1*, and *Dusp10* suggest a resting or quiescent phenotype, whereas expressions of cytokine receptors (*Il4r*, *Il7r*, *Il18r1*) implied that these cells could respond to cytokines (Figures 4c and 5d,e). Overall, trajectory analysis not only mapped the differentiation pathways of CD8 T cells in aged adipose tissue but also identified key regulatory genes like *Fcgr2b*, *Acvr2a*, *Il18r1*, whose expression dynamics may govern fate decisions within the CM/VM‐like CD8 T cells during aging.

### 2.6 Accumulation of Fcgr2b‐Expressing CM/VM‐Like CD8 T Cells in Aged gWAT Drives Inflammation

**FIGURE 6:**
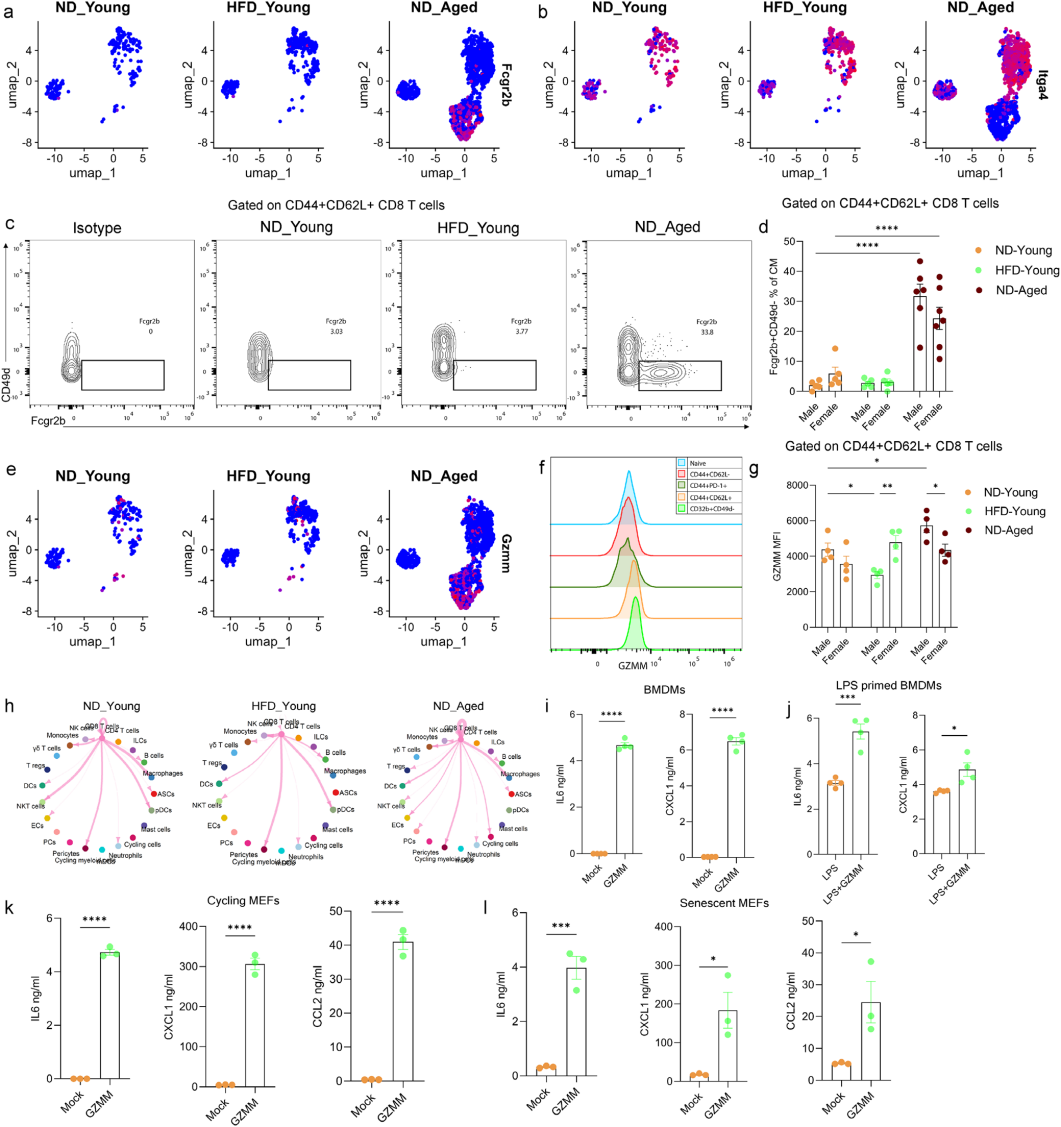
Accumulation of Fcgr2b‐expressing cells in the gWAT of aged mice. UMAP plot showing expression of (a) *Fcgr2b* and (b) *Itga4* in CD8 T cells from ND‐fed young, HFD‐fed young, ND‐fed aged mice. (c) Representative flow cytometry plots displaying the gating strategy for identifying Fcgr2b + CD49d‐ cells within CD44 + CD62L+ CD8 T cells. (d) Bar graph showing the frequency of Fcgr2b + CD49d‐ cells within CD44 + CD62L+ CD8 T cells (*N* = 5–7). (e) UMAP plot showing expression of *Gzmm* in CD8 T cells among different groups. (f) Histogram showing GZMM expression in CD8 T cell subsets measured using flow cytometry. (g) Bar plot representing the median fluorescent intensity (MFI) of GZMM in CD44 + CD62L+ CD8 T cells across different groups (*N* = 4). Statistical significance was determined using two‐way ANOVA followed by Tukey's HSD post hoc test. Only significant comparisons are shown between the sex of each group and with respect to ND‐fed young mice. (h) Circle plot depicting outgoing signals from CD8 T cells to other cell types in SVF based on L‐R expression analyzed using Cellchat. Each node represents a cell type, and edge width reflects interaction strength. Quantification of IL6 and CXCL1 levels in supernatants of (i) unprimed and (j) LPS‐primed bone marrow‐derived macrophages (BMDMs) following overnight stimulation with rGZMM, measured by ELISA. Quantification of IL6, CXCL1, and CCL2 in the supernatants of (k) cycling and (l) senescent mouse embryonic fibroblasts (MEFs) following stimulation with rGZMM for 48 h and 24 h, respectively, measured by ELISA. Statistical significance was determined using an unpaired *t*‐test. Data are presented as mean ± SEM. *p* values < 0.05 were considered significant. *p < 0.05, **p < 0.01, ***p < 0.001, ****p < 0.0001.

The inhibitory receptor *Fcgr2b* has been previously implicated in regulating CD8 T cells, with its loss being associated with an accumulation of effector cells (Morris, Farley, et al. 2020). In our dataset, pseudotime trajectory analysis suggested a regulatory role for *Fcgr2b* in CD8 T cell differentiation, with expression predominantly confined to CM/VM‐like (*Itga4*‐) CD8 T cells rather than EM cells (Figures 4b and 6a,b). Consistent with scRNA‐seq data, ND‐fed aged mice showed a significant increase in the frequency of Fcgr2b/CD32b + CD49d‐ cells within the CD44 + CD62L+ CD8+ T cells compared to both ND‐ and HFD‐fed young mice (Figure 6c,d and S5). Moreover, CM/VM‐like CD8+ T cells demonstrated elevated expression of GZMM relative to other CD8 T cell subsets (Figures 4c and 6e,f). Notably, aging was associated with a significant upregulation of GZMM, with ND‐fed aged males exhibited higher levels of GZMM compared to ND‐fed aged females, indicating sex‐specific differences in GZMM production (Figure 6g and S5). Ligand‐receptor analysis revealed that aged CD8 T cells could interact with multiple cell types, including macrophages and fibroblasts, via distinct signaling axes (Figure 6h and S7). Given the association between GZMM and inflammation (Shan et al. 2020; Baschuk et al. 2014; Anthony et al. 2010), we next explored its functional role in vitro on mouse macrophages and fibroblasts. Mouse bone marrow‐derived macrophages (BMDMs), both unprimed and LPS‐primed (Figure 6i,j), and cycling and senescent mouse embryonic fibroblasts (MEFs) (Figure 6i,k), were stimulated with recombinant mouse GZMM (rGZMM). In all cell types, rGZMM treatment significantly increased the secretion of pro‐inflammatory proteins, including IL‐6, CXCL1, and CCL2. These findings suggest that aged CD44 + CD62L + Fcgr2b + CD49d‐ CD8+ T cells, while retaining cytotoxic potential, may also contribute to adipose tissue inflammation via GZMM secretion. This expands the established paradigm of granzyme‐mediated inflammation, previously attributed to GZMK‐expressing exhausted CD8 T cells, suggesting that both GZMK and GZMM may mediate inflammation in the adipose tissue of aged mice through distinct CD8 T cell subsets (Mogilenko et al. 2021).

## 3 Discussion

Distinguishing the specific cellular changes associated with aging and obesity is essential for understanding their individual contributions to disease and for developing targeted interventions. Using a scRNA‐seq approach, we delineated the heterogeneity of VAT during physiological aging and HFD‐induced obesity. We observed that aging was associated not only with a marked accumulation of CD8 T cells in gWAT, but also with increased interaction strength between CD8 T cells and SVF cells, primarily mediated by MHC‐I and IL10 signaling. While both aging and obesity led to expansion of exhausted CD8 T cells, aging uniquely promoted accumulation of a phenotypically distinct population of CD8 T cells resembling VM‐like CD8 T cells. These cells expressed canonical memory‐associated markers *CD44*, *Sell*, *Il7r*, *Il2rb*, and exhibited reduced expression of *Itga4*, a VM phenotype. Notably, this subset also expressed *Fcgr2b*, *Acvr2a*, along with *Gzmm*, suggesting a potential role in immune regulation, cytotoxicity, and inflammation. These findings suggest that aging uniquely shapes the adipose tissue microenvironment to promote a VM‐like CD8 T cell phenotype with both regulatory and cytotoxic potential.

Both aged and obese mice exhibited increased signaling towards CD8 T cells. The majority of this signaling was mediated by engagement of MHC‐Ib molecules in both conditions. Although MHC‐I typically presents self‐peptides, the development of a senescent phenotype in adipose tissue cells during aging and obesity could explain the increased MHC‐I signaling compared to ND‐fed young mice (Zhang et al. 2024; Marin et al. 2023; Ou et al. 2022; Pereira et al. 2019; Palmer et al. 2019). MHC‐Ib molecules can interact with either inhibitory or activating receptors on CD8 T cells, and the outcome of this interaction likely shapes their functional response. In our dataset, CM/VM‐like CD8 T cells expressed high transcript levels of both inhibitory receptor Klrc1(NKG2A) and activating receptor Klrc2 (NKG2C). Given that NKG2A exhibits higher binding affinity to MHC‐Ib molecules than NKG2C, these interactions may favor inhibitory signaling and functional suppression of CD8 T cells (Wu et al. 2023; Pereira et al. 2019; Béziat et al. 2011). Notably, most cell types, such as B cells, DCs, ILCs, Tregs, γδT cells, PCs, etc., in the gWAT of aged and obese mice exhibited increased MHC‐I signaling compared to young controls, with the exception of Pdgfra‐expressing ASCs. This suggests that ASCs may be vulnerable to CD8 T cell‐mediated cytotoxicity, although this requires further validation.

In addition to MHC‐I‐mediated signaling, CD8 T cells in gWAT also received signals via *Cd274* (PD‐L1), *Il10*, *Bmp4/6*, and *Inhbb* ligands. Correspondingly, elevated expression of their receptors, *Epha3*, *Il10ra*, and *Acvr2a* were observed in CD8 T cells of aged mice. Both PD‐L1 and IL10 are known to induce an immunosuppressive state in CD8 T cells (Cha et al. 2019; Smith et al. 2018). In our analysis, *Epha3* and *Il10ra* were selectively expressed in effector and exhausted CD8 T cell subsets, supporting their role in promoting T cell dysfunction. Although the role of *Epha3* in CD8 T cells is not well defined, it has been reported to be upregulated in malignant T cells (Maddigan et al. 2011; Smith et al. 2004).

Notably, we found that *Acvr2a* was highly expressed in the *Klrc1*‐expressing CM/VM‐like CD8 T cell cluster. As *Acvr2a* can signal through both SMAD1/5 and SMAD2/3 pathways, the relative balance of *Bmp4*, *Inhbb*, and *Tgfb1* signaling in the aged adipose tissue microenvironment may influence CD8 T cell fate by either promoting a dysfunctional state or inducing stemness (Hu et al. 2025; Saadey et al. 2023; Pinjusic et al. 2022; Appiah Adu‐Gyamfi et al. 2020; Olsen et al. 2015). Increased frequencies of VM CD8 T cells during aging have been well documented in the peripheral blood, spleen, and lymph nodes (Davenport et al. 2019; Quinn et al. 2018, 2020; Chiu et al. 2013; Hussain et al. 2023; Borsa et al. 2021; Hussain and Quinn 2019). In our dataset, we have further demonstrated their accumulation in gWAT of aged mice. Both CM and VM share multiple features including expression of *Sell* (CD62L), *Cd44*, *Il7r*, *Eomes*, but can be distinguished by CD49d, which is absent on VM CD8 T cells (Hussain and Quinn 2019). Accordingly, we identified VM CD8 T cells as CD122 + CD49d‐ within the CD44 + CD62L+ CD8+ T cell population. The accumulation of CD49d‐ VM‐like CD8 T cells in aged adipose tissue may be driven by cytokine‐mediated homeostatic expansion (Quinn et al. 2018; Chiu et al. 2013; Renkema et al. 2014).

Studies from the past decade have demonstrated the *Fcgr2b* expression on memory CD8 T cells, contrary to the belief that it is expressed only on B cells and on innate immune cells (Morris, Pinelli, et al. 2020; Morris, Farley, et al. 2020; Starbeck‐Miller et al. 2014; Nimmerjahn and Ravetch 2008). In the present study, we demonstrated higher expression levels of *Acvr2a* and *Fcgr2b* in CM/VM‐like CD8 T cells of aged mice. Fcgr2b is the only inhibitory Fc receptor that signals through an immunoreceptor tyrosine based inhibitory motif (ITIM) in its cytoplasmic domain (Getahun and Cambier 2015). Morris et al. reported that *Fcgr2b* is predominantly expressed by effector memory (CD44 + CD62Llo) CD8 T cells, and its engagement by Fgl2 can induce apoptosis and limit their expansion (Morris, Farley, et al. 2020). Microbial stimulation has also been shown to induce Fcgr2b expression on memory CD8 T cells (Morris, Pinelli, et al. 2020; Starbeck‐Miller et al. 2014), its engagement by preexisting antigen–antibody complexes has been reported to limit expansion of memory cells during homologous rechallenge (Starbeck‐Miller et al. 2014). Importantly, elevated *Fcgr2b* expression has been observed on CD8+ tumor‐infiltrating lymphocytes in patients with myeloma and in patients with SARS‐CoV‐2 (Morris et al. 2025; Bennion et al. 2023; Baecher and Ford 2021). Additional studies have suggested that *Fcgr2b* expression on effector CD8 T cells impairs responsiveness to anti‐PD‐1 therapy, whereas its deletion enhances CD8 T cell stemness (Ku et al. 2024; Bennion et al. 2023). More recently, *Fcgr2b* has been detected on aged regulatory CD8 T cells, suggesting broader immunoregulatory functions during aging (Srinivasan et al. 2025).

In our scRNA‐seq data, *Fcgr2b* transcripts were enriched in VM‐like CD8 cells compared to EM CD8 T cells. Expression of *Fcgr2b*, as well as the proportion of *Fcgr2b*‐expressing cells, was highest in the TGzmm cluster. Flow cytometry further confirmed age‐related accumulation of Fcgr2b + CD49d‐ CD8 T cells in gWAT. Moreover, age‐associated accumulation of IgG, a *Fcgr2b* ligand, has been reported in visceral adipose tissue (Yu et al. 2024). While engagement of IgG‐Fcgr2b could theoretically promote apoptosis, these cells expressed high levels of Bcl2, suggesting a mechanism for survival under proapoptotic pressure. Interestingly, these cells also expressed Fcgrt, the neonatal Fc receptor, further supporting a role of IgG in modulating CD8 T cell function in gWAT of aged mice. RNA velocity indicated that *Fcgr2b*‐expressing TGzmm cells may differentiate into dysfunctional state (Tcm/cm2), suggesting the potential engagement of Fcgr2b in aged mice. Age‐related accumulation of dysfunctional VM CD8 T cells has been identified in mice and humans which could blunt CD8 T cell responses (Quinn et al. 2018) and increase susceptibility to tumors and infections. Fcgr2b on EM CD8 T cells has already been identified as a promising target to improve checkpoint inhibitory therapy against tumors (Bennion et al. 2023; Baecher and Ford 2021). Our pseudotime trajectory analysis suggests that Fcgr2b may regulate CD8 T cell fate decisions; however, functional studies are needed to establish its role in VM‐like CD8 T cells during aging and age‐related diseases in humans. Consistent with the previous report, we have also observed that *Fcgr2b*‐expressing cluster exhibited a cytotoxic profile (Bennion et al. 2023) These data suggest that the *Fcgr2b*‐expressing TGzmm cluster represents a transitional cell state capable of inducing cytotoxicity and granzyme M production.

The accumulation of CD8 T cells has been linked to adipose tissue inflammation (Nishimura et al. 2009). More recently, age‐associated *Gzmk*‐expressing CD8 T cells (Taa) have been shown to induce inflammatory responses in fibroblast cells (Mogilenko et al. 2021). In our dataset, *Gzmk* expression was elevated in CD8 T cells from both aged and obese mice, while *Gzmm* expression was selectively increased in aged mouse CD8 T cells. Although granzymes are well known to mediate cytotoxicity, emerging evidence suggests that they can also regulate inflammation (Aubert et al. 2024; Zheng et al. 2023; Shan et al. 2020; Baschuk et al. 2014; Anthony et al. 2010). We found that GZMM treatment was sufficient to induce proinflammatory cytokine release from both mouse fibroblasts and macrophages. Elevated GZMM levels, as well as GZMM positive CD8 T cells, have been reported in the synovial fluids of rheumatoid arthritis patients, further supporting its role in human inflammatory conditions (Shan et al. 2020). The accumulation of these inflammatory mediators during aging likely contributes to adipose tissue dysfunction, and their identification could help in the development of targeted therapies for age‐related diseases. Although *Gzmk* expressing cells make up a large fraction of CD8 T cells (~50% proportion of CD8 T cells, including Tem, Tex1, and Tex2 clusters) which may induce inflammation in aged adipose tissue, *Gzmm* expressing cells (~20% TGzmm cluster) could further exacerbate the inflammatory burden. Collectively, we have shown that VM‐like CD8 T cells accumulate in visceral adipose tissue during aging. Their differentiation trajectory appears to involve signaling through *Acvr2a* and *Fcgr2b*. During the transition towards dysfunctional states, these cells acquire cytotoxic potential, marked by *Gzmm* expression, which may contribute to cytotoxicity and adipose tissue inflammation. Future studies will be required to determine the specific role of these cells in regulating cytotoxicity and inflammation in aged adipose tissue.

This study has some limitations. First, in the current study, we used a standard normal/chow diet rather than a purified low‐fat diet (LFD) in comparison to the HFD diet. However, the use of a purified LFD on aged mice is time‐consuming, and its prolonged exposure may itself alter normal physiological changes in visceral adipose tissue by affecting metabolic processes. We were unable to validate Acvr2a expression using flow cytometry because of the lack of a specific monoclonal antibody suitable for flow cytometry. However, we observed high levels of *Acvr2a* transcripts in CM/VM‐like CD8 T cell clusters, suggesting the potential significance of this receptor on CD8 T cells during aging. We did not utilize *Fcgr2b* knockout mice to directly assess the functional significance of *Fcgr2b*‐expressing cells in aging or their role in modulating CD8 T cell fate. However, our findings highlight the presence of *Fcgr2b*‐expressing VM CD8 T cells in aged mice and their potential functional significance. Lastly, we did not evaluate the presence of these VM‐like CD8 T cells in other organs during aging because the primary focus of the current study was to investigate the differences in visceral adipose tissue during diet‐induced obesity and physiological aging. Future studies are needed to elucidate the function of these cells in the context of aging and age‐related diseases.

## 4 Conclusion

Our study provides a comprehensive single‐cell analysis of SVF isolated from gWAT of aged and obese mice of both sexes. We highlighted key differences in the cellular heterogeneity of gWAT between aging and obesity. Using cell–cell interaction and trajectory analysis, we identified that *Acvr2a* and *Fcgr2b* may modulate CD8 T cell differentiation in aged adipose tissue. We further show that *Fcgr2b*‐expressing cells exhibit a cytotoxic profile and may contribute to Gzmm‐dependent inflammatory signaling. These findings provide new insights into VM‐like CD8 T cells in aged adipose tissue and suggest *Fcgr2b* as a potential modulator of their differentiation and functional state during aging.

## 5 Methods

### 5.1 Mice and Diet

C57BL/6J male and female mice (young: 1–2 months; aged: 18–21 months) were obtained from Jackson Laboratory. Mice were housed in a specific pathogen‐free (SPF) facility at the University of Michigan. Young mice were fed a high‐fat diet (HFD) (42% Kcal from fat, Inotiv; TD.88137) for an additional 12 weeks, while the rest of the young and aged mice were maintained on a normal/chow diet (ND) (13% Kcal from fat, LabDiet; 5L0D) for the same time period. No microbiota normalization procedures were performed to maintain the natural differences in the microbiota among ND‐fed young, HFD‐fed young, and ND‐fed aged mice. All experiments were approved by the Unit of Laboratory Animal Medicine, University of Michigan, under animal protocols PRO00010459 and PRO00012394 and were performed accordingly.

### 5.2 Glucose and Insulin Tolerance Tests (GTT and ITT)

For glucose tolerance tests (GTT), mice were fasted overnight and intraperitoneally (i.p.) injected with glucose at a dose of 2.0 g/kg body weight. For insulin tolerance tests (ITT), mice were fasted for 6 h prior to i.p. injection of Humulin R (Eli Lilly and Co.) at 0.8 U/kg body weight. Blood glucose levels were measured using the Clarity BG1000 blood glucose monitoring system (Clarity Diagnostics) at baseline and at intervals of 15–30 min for up to 2 h postinjection.

### 5.3 Stromal Vascular Fraction (SVF) Isolation

Mice were euthanized using CO_2_ inhalation, and gonadal white adipose tissue (gWAT) was dissected. Tissue was finely minced and digested in 0.8 mg/mL Collagenase II (Worthington Biochemical) buffer containing 3% BSA, 1X penicillin/streptomycin, 1.2 mM CaCl₂, 1 mM MgCl₂, 0.8 mM ZnCl₂, and 15 mM HEPES for 40 min at 37°C with constant agitation. The digested suspension was centrifuged at 600 g for 10 min at 4°C, and the SVF was filtered through 70 μm and 40 μm strainers. Red blood cell (RBC) lysis was performed as per the manufacturer's instructions (eBioscience), followed by washing and resuspension in RPMI 1640 supplemented with 10% FBS and 1X penicillin/streptomycin. Fresh cells were processed for single cell RNA sequencing, and the remaining cells were cryopreserved.

### 5.4 Single‐Cell RNA Sequencing (scRNA‐Seq)

SVF cells were stained with Zombie Aqua live/dead dye (Biolegend) for 10 min at room temperature. Cells were sorted using a Bigfoot Spectral Cell Sorter (Thermo Fisher Scientific). Sorted cells were resuspended in RPMI medium with 10% FBS; equal numbers of cells per group per sex were pooled and submitted to the University of Michigan Advanced Genomics Core for 3′ library preparation and sequencing. Briefly, cell counts were obtained using the Luna‐FX7 Cell Counter (LogosBio). Libraries were prepared using the 10× Genomics Chromium Controller with 3′ v3.1 chemistry and Feature Barcoding technology for Cell Multiplexing, according to the manufacturer's instructions (10× Genomics). Library quality was assessed on the LabChip GXII HT (PerkinElmer), and concentrations were determined using Qubit (Thermo Fisher). Pooled libraries were sequenced using paired‐end 28 × 10 × 10 × 151 bp reads on the Illumina NovaSeq XPlus. Demultiplexed FASTQ files were generated using Bcl2fastq2 (Illumina), and count matrices were generated using the CellRanger pipeline (10× Genomics) (Zheng et al. 2017).

### 5.5 scRNA‐Seq Analysis

Data were analyzed using Seurat v 5.1.0 (Butler et al. 2018). Cells were filtered to retain those with ≥ 500 UMIs, ≥ 250 genes, log10(genes per UMI) > 0.8, and mitochondrial gene content < 15%. Genes expressed in fewer than 10 cells were excluded. Doublets were detected using scDblFinder v1.16.0 (Germain et al. 2022) and further removed. Each dataset was normalized, and 4000 variable features were identified using the “vst” method. Integration was performed using SelectIntegrationFeatures, FindIntegrationAnchors, and IntegrateData. Variables including gene count, mitochondrial ratio, and cell cycle scores (S and G2M) were regressed during scaling. PCA was performed using 60 principal components (PCs). UMAP was applied on top 40 PCs, guided by elbow plot analysis. Clustering was performed at a resolution of 1.2. Cell clusters were annotated using SingleR v2.4.1, referencing the ImmGen and MouseRNAseq datasets from the cellDex v1.12.0 package (Aran et al. 2019). Annotations were finally refined manually based on top marker genes identified via FindAllMarkers.

### 5.6 Cell–Cell Communication Analysis

Intercellular communication in the adipose SVF was analyzed using CellChat v2.1.2 and MultiNicheNet v2.0.1 (Jin et al. 2025; Browaeys et al. 2023). Clusters with < 10 cells in any group were removed from the analysis. Ligand‐receptor (L‐R) interactions were analyzed across various cell types by calculating communication probabilities between different cells using computeCommunProb(), and filtering out communication involving < 10 cells using filterCommunication().

For focused analysis of signaling input to CD8 T cells, MultiNicheNet was employed to identify L‐R pairs based on differentially expressed genes (DEGs) from multigroup data. Cell types with < 10 cells per group were excluded, and DEGs with log2 fold change ≥ 0.5 and adjusted *p* value ≤ 0.05 were used to define the top 100 L‐R interactions across conditions.

### 5.7 CD8 T Cell Subset Analysis

CD8a‐expressing cells were subsetted into a new Seurat object and subjected to standard preprocessing and clustering as described earlier. UMAP dimensionality reduction was performed using 15 PCs, and clusters were resolved at a resolution of 0.6. CD8 T cell clusters were manually annotated based on the expression of canonical markers. Naïve = (*Ccr7*+ *Lef1*+ *IL7r* + *Sell* + *Cd44*‐ *Ccl5*‐), Tcm/vm‐like cells (*Ccr7*+ *Lef1*+ *IL7r* + *Sell* + *Cd44*+ *Ccl5*+ *Il2rb* + *Il18r1*+ *Itga4*‐), Tem (*Cd44*+ *Sell*‐ *Tcf7*+ *IL7r* + *Ccl5*+), Tex (*Pdcd1*+ *Tox* + *Nr4a2*+ *Nr3c1*+), Teff (*Tbx21*+ *Zeb2*+ *Gzmb* + *Prf1*+ *Klrg1*+). Markers' expression was visualized using the Dotplot function, and cluster proportions were calculated relative to the total CD8 T cells. Enrichment analysis was performed on differentially expressed genes from the Tcm/vm2 cluster using the clusterProfiler (4.10.1) (Yu et al. 2012). Gene Ontology (GO) was performed using the function enrichGO(), and the top 10 significant GO terms were visualized using cnetplot(). Gene set enrichment analysis (GSEA) was performed using Hallmark pathways from MSigDB using msigdbr (10.0.1).

### 5.8 RNA Velocity and Monocle Pseudotime Analysis

RNA velocity analysis was performed using Velocyto (v0.17.17) and scVelo (v0.3.3) (La Manno et al. 2018; Bergen et al. 2020). Individual loom files were generated from the CellRanger output using velocyto and subsequently merged using loompy.combine(). The combined loom file was loaded into Scanpy (v1.11.0) (Wolf et al. 2018), and CD8 T cells were subsetted for further analysis using metadata, cell barcodes, and UMAP coordinates exported from Seurat. RNA velocity was computed using scv.tl.velocity(mode = “stochastic”) and visualized on CD8 UMAP embeddings using scv.pl.velocity_embedding_stream, with cells colored by CD8 clusters.

Trajectory inference was performed using Monocle3 (1.3.7) (Trapnell et al. 2014). Seurat objects were converted into cell_data_set format, and size factors were estimated with estimate_size_factors(). Cells were clustered, and trajectories were learned using learn_graph(). Root cells were defined as naïve CD8 T cells, and pseudotime was assigned via order_cells(). Cells were visualized by pseudotime using plot_cells(). To identify genes dynamically expressed along pseudotime, we used the graph_test() function with the principal_graph. The top 20 pseudotime‐associated genes were visualized in a heatmap using pheatmap (1.0.12), with cells ordered by pseudotime and annotated by CD8 cluster identity.

### 5.9 Flow Cytometry

Cryopreserved SVF cells were thawed, and 0.5–2 million cells per sample were used for staining. Cells were washed and stained for live/dead staining using Zombie Aqua (Biolegend). Fc receptor blocking was performed using TruStain FcX (antimouse CD16/32, Biolegend) antibody according to the manufacturer's instructions, except for samples stained for Fcgr2b/CD32b, where Fc blocking was omitted. Surface staining was carried out at 4°C for 30 min. Following surface staining, cells were washed, fixed, and permeabilized using eBioscience Foxp3 staining buffer (Thermo) and stained for intracellular proteins. Data acquisition was performed on ID7000 (Sony), and the unmixed FCS files were analyzed using FlowJo v10.10.0 (FlowJoLLC). Antimouse BV421 CD69 (H1.2F3), BV605 CD44 (IM7), BV711 CD62L (MEL‐14), BV785 NK1.1 (PK136), BV785 CD19 (6D5), BV785 F4/80 (BM8), BV785 CD14 (Sa14‐2), BV785 CD11c (N418), AF488 CD127 (A7R34), AF700 CD4 (RM4‐5), APC‐Fire750 PD‐1 (29F.1A12), APC‐Fire810 CD8 (53–6.7) were purchased from Biolegend; BUV395 CD3 (17A2), BUV496 CD45 (30‐F11), BUV737 CD49d (9C10[MFR4.B]), BB700 CD122 (TM‐β1) were purchased from BD Biosciences; PE Tox (TXRX10), PE Fcgr2b/CD32b (AT130‐2), PE‐eFluor610 Eomes (Dan11mag) were purchased from Thermo; AF647 TCF1/TCF7 (C63D9) from Cell Signaling and APC GZMM (MBS2042310) from MyBioSource.

### 5.10 Granzyme M In Vitro Stimulation

Bone marrow‐derived macrophages (BMDMs) were thawed and rested overnight in DMEM supplemented with 10% FCS and 20% L929‐conditioned media. Mouse recombinant granzyme M (rGZMM; MyBioSource) was reconstituted in a buffer containing 20 mM Tris and 150 mM NaCl (pH 8.0) according to the manufacturer's instructions. Cells treated with reconstitution buffer alone (no GZMM) were considered as mock. BMDMs were then primed with lipopolysaccharide (LPS, 10 ng/mL) for 3 h at 37°C. Both primed and unprimed BMDMs were washed and subsequently stimulated with rGZMM (100 ng/mL) in 2% FCS and 20% L929‐conditioned media for 24 h at 37°C.

Senescence was induced in mouse embryonic fibroblasts (MEFs) as described previously (Mogilenko et al. 2021). Briefly, MEFs were cultured in 10% FCS supplemented DMEM up to 70% confluency, followed by treatment with 0.1 μM doxorubicin for 24 h. The media were then replaced with fresh DMEM, and cells were cultured for an additional 24 h before being treated again with 0.1 μM doxorubicin (Sigma) for another 24 h. MEFs were then incubated in fresh media for 7 days for senescence induction. Both cycling and senescent cells were stained using the β‐gal activity assay kit as per the manufacturer's instructions (Cell Signaling) (Figure S9). Cycling and senescent MEFs were subsequently treated with rGZMM (100 ng/mL) in DMEM without FCS for 48 h and 24 h, respectively. Supernatants collected from BMDMs and MEFs were used for quantitative estimation of IL6, CXCL1, and CCL2 by DuoSet ELISA kits, following the manufacturer's instructions (R&D Systems).

### 5.11 Statistical Analysis

Data are presented as mean ± standard error of the mean (SEM). Statistical significance between groups and sexes was assessed using two‐way ANOVA followed by Tukey's HSD post hoc test. An unpaired *t*‐test was used to compare rGZMM‐treated and untreated samples. *p* values < 0.05 were considered significant. Significance levels are indicated in the figure legends as follows: *p < 0.05, **p < 0.01, ***p < 0.001, ****p < 0.0001.

## Author Contributions

Study design: A.K. and R.Y. Experiments: A.K. and M.O'B. Data acquisition and analysis: A.K. Writing: A.K. Reviewing and editing: A.K., R.Y., and V.B.Y. Funding: R.Y. and V.B.Y.

## Conflicts of Interest

The authors declare no conflicts of interest.

## Supporting information


**Appendix S1:** acel70278‐sup‐0001‐AppendixS1.pdf.

## Data Availability

The data that support the findings of this study are openly available in Gene Expression Omnibus at https://www.ncbi.nlm.nih.gov/geo/query/acc.cgi?acc=GSE300036, reference number GSE300036.

## References

[acel70278-bib-0001] Anthony, D. A. , D. M. Andrews , M. Chow , et al. 2010. “A Role for Granzyme M in TLR4‐Driven Inflammation and Endotoxicosis.” Journal of Immunology 185: 1794–1803. 10.4049/jimmunol.1000430. 20585036

[acel70278-bib-0002] Appiah Adu‐Gyamfi, E. , F. Tanam Djankpa , W. Nelson , et al. 2020. “Activin and Inhibin Signaling: From Regulation of Physiology to Involvement in the Pathology of the Female Reproductive System.” Cytokine 133: 155105. 10.1016/j.cyto.2020.155105. 32438278

[acel70278-bib-0003] Aran, D. , A. P. Looney , L. Liu , et al. 2019. “Reference‐Based Analysis of Lung Single‐Cell Sequencing Reveals a Transitional Profibrotic Macrophage.” Nature Immunology 20: 163–172. 10.1038/s41590-018-0276-y. 30643263 PMC6340744

[acel70278-bib-0004] Aubert, A. , K. Jung , S. Hiroyasu , J. Pardo , and D. J. Granville . 2024. “Granzyme Serine Proteases in Inflammation and Rheumatic Diseases.” Nature Reviews Rheumatology 20: 361–376. 10.1038/s41584-024-01109-5. 38689140

[acel70278-bib-0005] Baecher, K. M. , and M. L. Ford . 2021. “Intersection of FcγRIIB, the Microbiome, and Checkpoint Inhibitors in Antitumor Immunity.” Cancer Immunology, Immunotherapy 70: 3397–3404. 10.1007/s00262-021-03004-4. 34241677 PMC10992943

[acel70278-bib-0006] Bapat, S. P. , J. Myoung Suh , S. Fang , et al. 2015. “Depletion of Fat‐Resident Treg Cells Prevents Age‐Associated Insulin Resistance.” Nature 528: 137–141. 10.1038/nature16151. 26580014 PMC4670283

[acel70278-bib-0007] Baschuk, N. , N. Wang , S. V. Watt , et al. 2014. “NK Cell Intrinsic Regulation of MIP‐1α by Granzyme M.” Cell Death & Disease 5: e1115. 10.1038/cddis.2014.74. 24625974 PMC3973215

[acel70278-bib-0008] Bénézech, C. , N.‐T. Luu , J. A. Walker , et al. 2015. “Inflammation‐Induced Formation of Fat‐Associated Lymphoid Clusters.” Nature Immunology 16: 819–828. 10.1038/ni.3215. 26147686 PMC4512620

[acel70278-bib-0009] Bennion, K. B. , M. Tariq , M. M. Wyatt , et al. 2023. “FcγRIIB Expressed on CD8+ T Cells Limits Responsiveness to PD‐1 Checkpoint Inhibition in Cancer.” Science Translational Medicine 15: eadd1868. 10.1126/scitranslmed.add1868. 37611081 PMC11325091

[acel70278-bib-0010] Bergen, V. , M. Lange , S. Peidli , F. A. Wolf , and F. J. Theis . 2020. “Generalizing RNA Velocity to Transient Cell States Through Dynamical Modeling.” Nature Biotechnology 38: 1408–1414. 10.1038/s41587-020-0591-3. 32747759

[acel70278-bib-0011] Béziat, V. , B. Hervier , A. Achour , D. Boutolleau , A. Marfain‐Koka , and V. Vieillard . 2011. “Human NKG2A Overrides NKG2C Effector Functions to Prevent Autoreactivity of NK Cells.” Blood 117: 4394–4396. 10.1182/blood-2010-11-319194. 21511964

[acel70278-bib-0012] Borsa, M. , N. Barandun , F. Gräbnitz , et al. 2021. “Asymmetric Cell Division Shapes Naive and Virtual Memory T‐Cell Immunity During Ageing.” Nature Communications 12: 2715. 10.1038/s41467-021-22954-y. PMC8113513 33976157

[acel70278-bib-0013] Bouneaud, C. , Z. Garcia , P. Kourilsky , and C. Pannetier . 2005. “Lineage Relationships, Homeostasis, and Recall Capacities of Central‐ and Effector‐Memory CD8 T Cells In Vivo.” Journal of Experimental Medicine 201: 579–590. 10.1084/jem.20040876. 15710650 PMC2213051

[acel70278-bib-0014] Browaeys, R. , J. Gilis , C. Sang‐Aram , et al. 2023. “MultiNicheNet: A Flexible Framework for Differential Cell‐Cell Communication Analysis From Multi‐Sample Multi‐Condition Single‐Cell Transcriptomics Data.” http://biorxiv.org/lookup/doi/10.1101/2023.06.13.544751.

[acel70278-bib-0015] Bruno, M. E. C. , S. Mukherjee , W. L. Powell , et al. 2022. “Accumulation of γδ T Cells in Visceral Fat With Aging Promotes Chronic Inflammation.” Geroscience 44: 1761–1778. 10.1007/s11357-022-00572-w. 35477832 PMC9213615

[acel70278-bib-0016] Butler, A. , P. Hoffman , P. Smibert , E. Papalexi , and R. Satija . 2018. “Integrating Single‐Cell Transcriptomic Data Across Different Conditions, Technologies, and Species.” Nature Biotechnology 36: 411–420. 10.1038/nbt.4096. PMC6700744 29608179

[acel70278-bib-0017] Camell, C. D. 2022. “Adipose Tissue Microenvironments During Aging: Effects on Stimulated Lipolysis.” Biochimica et Biophysica Acta ‐ Molecular and Cell Biology of Lipids 1867: 159118. 10.1016/j.bbalip.2022.159118. 35131468 PMC8986088

[acel70278-bib-0018] Camell, C. D. , P. Günther , A. Lee , et al. 2019. “Aging Induces an Nlrp3 Inflammasome‐Dependent Expansion of Adipose B Cells That Impairs Metabolic Homeostasis.” Cell Metabolism 30: 1024–1039.e6. 10.1016/j.cmet.2019.10.006. 31735593 PMC6944439

[acel70278-bib-0019] Cha, J.‐H. , L.‐C. Chan , C.‐W. Li , J. L. Hsu , and M.‐C. Hung . 2019. “Mechanisms Controlling PD‐L1 Expression in Cancer.” Molecular Cell 76: 359–370. 10.1016/j.molcel.2019.09.030. 31668929 PMC6981282

[acel70278-bib-0020] Chiu, B.‐C. , B. E. Martin , V. R. Stolberg , and S. W. Chensue . 2013. “Cutting Edge: Central Memory CD8 T Cells in Aged Mice Are Virtual Memory Cells.” Journal of Immunology 191: 5793–5796. 10.4049/jimmunol.1302509. PMC3858473 24227783

[acel70278-bib-0021] Clambey, E. T. , J. White , J. W. Kappler , and P. Marrack . 2008. “Identification of Two Major Types of Age‐Associated CD8 Clonal Expansions With Highly Divergent Properties.” Proceedings of the National Academy of Sciences of the United States of America 105: 12997–13002. 10.1073/pnas.0805465105. 18728183 PMC2525558

[acel70278-bib-0022] Cottam, M. A. , H. L. Caslin , N. C. Winn , and A. H. Hasty . 2022. “Multiomics Reveals Persistence of Obesity‐Associated Immune Cell Phenotypes in Adipose Tissue During Weight Loss and Weight Regain in Mice.” Nature Communications 13: 2950. 10.1038/s41467-022-30646-4. PMC9135744 35618862

[acel70278-bib-0023] Davenport, B. , J. Eberlein , V. van der Heide , et al. 2019. “Aging of Antiviral CD8+ Memory T Cells Fosters Increased Survival, Metabolic Adaptations, and Lymphoid Tissue Homing.” Journal of Immunology 202: 460–475. 10.4049/jimmunol.1801277. PMC6358025 30552164

[acel70278-bib-0024] Emont, M. P. , C. Jacobs , A. L. Essene , et al. 2023. “Author Correction: A Single‐Cell Atlas of Human and Mouse White Adipose Tissue.” Nature 620: E14. 10.1038/s41586-023-06445-2. 37495702

[acel70278-bib-0025] Ferrucci, L. , and E. Fabbri . 2018. “Inflammageing: Chronic Inflammation in Ageing, Cardiovascular Disease, and Frailty.” Nature Reviews. Cardiology 15: 505–522. 10.1038/s41569-018-0064-2. 30065258 PMC6146930

[acel70278-bib-0026] Franceschi, C. , P. Garagnani , P. Parini , C. Giuliani , and A. Santoro . 2018. “Inflammaging: A New Immune‐Metabolic Viewpoint for Age‐Related Diseases.” Nature Reviews. Endocrinology 14: 576–590. 10.1038/s41574-018-0059-4. 30046148

[acel70278-bib-0027] Geginat, J. , A. Lanzavecchia , and F. Sallusto . 2003. “Proliferation and Differentiation Potential of Human CD8+ Memory T‐Cell Subsets in Response to Antigen or Homeostatic Cytokines.” Blood 101: 4260–4266. 10.1182/blood-2002-11-3577. 12576317

[acel70278-bib-0028] Geginat, J. , F. Sallusto , and A. Lanzavecchia . 2001. “Cytokine‐Driven Proliferation and Differentiation of Human Naive, Central Memory, and Effector Memory CD4(+) T Cells.” Journal of Experimental Medicine 194: 1711–1719. 10.1084/jem.194.12.1711. 11748273 PMC2193568

[acel70278-bib-0029] Germain, P.‐L. , A. Lun , C. Garcia Meixide , W. Macnair , and M. D. Robinson . 2022. “Doublet Identification in Single‐Cell Sequencing Data Using scDblFinder.” F1000Research 10: 979. 10.12688/f1000research.73600.2. PMC9204188 35814628

[acel70278-bib-0030] Getahun, A. , and J. C. Cambier . 2015. “Of ITIMs, ITAMs, and ITAMis: Revisiting Immunoglobulin Fc Receptor Signaling.” Immunological Reviews 268: 66–73. 10.1111/imr.12336. 26497513 PMC4621791

[acel70278-bib-0031] Goronzy, J. J. , F. Fang , M. M. Cavanagh , Q. Qi , and C. M. Weyand . 2015. “Naive T Cell Maintenance and Function in Human Aging.” Journal of Immunology 194: 4073–4080. 10.4049/jimmunol.1500046. PMC4452284 25888703

[acel70278-bib-0032] Hu, L. , Y. Zhao , X. Zhang , and C. Ma . 2025. “Activin A Promoted the Anti‐Tumor Effect of ActRIIA High CD8+ T Cells in Mouse Hepatoma.” Cancer Medicine 14: e70147. 10.1002/cam4.70147. 39727149 PMC11672029

[acel70278-bib-0033] Hussain, T. , A. Nguyen , C. Daunt , et al. 2023. “Helminth Infection‐Induced Increase in Virtual Memory CD8 T Cells Is Transient, Driven by IL‐15, and Absent in Aged Mice.” Journal of Immunology (Baltimore, Md.: 1950) 210: 297–309. 10.4049/jimmunol.2200316. 36524995

[acel70278-bib-0034] Hussain, T. , and K. M. Quinn . 2019. “Similar but Different: Virtual Memory CD8 T Cells as a Memory‐Like Cell Population.” Immunology and Cell Biology 97: 675–684. 10.1111/imcb.12277. 31140625

[acel70278-bib-0035] Jin, S. , M. V. Plikus , and Q. Nie . 2025. “CellChat for Systematic Analysis of Cell‐Cell Communication From Single‐Cell Transcriptomics.” Nature Protocols 20: 180–219. 10.1038/s41596-024-01045-4. 39289562

[acel70278-bib-0036] Kar, A. , M. Alvarez , K. M. Garske , et al. 2024. “Age‐Dependent Genes in Adipose Stem and Precursor Cells Affect Regulation of Fat Cell Differentiation and Link Aging to Obesity via Cellular and Genetic Interactions.” Genome Medicine 16: 19. 10.1186/s13073-024-01291-x. 38297378 PMC10829214

[acel70278-bib-0037] Kohlgruber, A. C. , S. T. Gal‐Oz , N. M. LaMarche , et al. 2018. “γδ T Cells Producing Interleukin‐17A Regulate Adipose Regulatory T Cell Homeostasis and Thermogenesis.” Nature Immunology 19: 464–474. 10.1038/s41590-018-0094-2. 29670241 PMC8299914

[acel70278-bib-0038] Ku, K. B. , C. W. Kim , Y. Kim , et al. 2024. “Inhibitory Fcγ Receptor Deletion Enhances CD8 T Cell Stemness Increasing Anti‐PD‐1 Therapy Responsiveness Against Glioblastoma.” Journal for Immunotherapy of Cancer 12: e009449. 10.1136/jitc-2024-009449. 39461881 PMC11529582

[acel70278-bib-0039] La Manno, G. , R. Soldatov , A. Zeisel , et al. 2018. “RNA Velocity of Single Cells.” Nature 560: 494–498. 10.1038/s41586-018-0414-6. 30089906 PMC6130801

[acel70278-bib-0040] Leonardi, G. C. , G. Accardi , R. Monastero , F. Nicoletti , and M. Libra . 2018. “Ageing: From Inflammation to Cancer.” Immunity & Ageing 15: 1. 10.1186/s12979-017-0112-5. 29387133 PMC5775596

[acel70278-bib-0041] Liao, X. , Q. Zeng , L. Xie , et al. 2024. “Adipose Stem Cells Control Obesity‐Induced T Cell Infiltration Into Adipose Tissue.” Cell Reports 43: 113963. 10.1016/j.celrep.2024.113963. 38492218

[acel70278-bib-0042] Lumeng, C. N. , J. Liu , L. Geletka , et al. 2011. “Aging Is Associated With an Increase in T Cells and Inflammatory Macrophages in Visceral Adipose Tissue.” Journal of Immunology 187: 6208–6216. 10.4049/jimmunol.1102188. PMC3237772 22075699

[acel70278-bib-0043] Maddigan, A. , L. Truitt , R. Arsenault , et al. 2011. “EphB Receptors Trigger Akt Activation and Suppress Fas Receptor‐Induced Apoptosis in Malignant T Lymphocytes.” Journal of Immunology 187: 5983–5994. 10.4049/jimmunol.1003482. 22039307

[acel70278-bib-0044] Marin, I. , M. Serrano , and F. Pietrocola . 2023. “Recent Insights Into the Crosstalk Between Senescent Cells and CD8 T Lymphocytes.” NPJ Aging 9: 8. 10.1038/s41514-023-00105-5. 37015935 PMC10073090

[acel70278-bib-0045] Medina‐Urrutia, A. X. , N. E. Antonio‐Villa , F. D. Martínez‐Sánchez , et al. 2025. “Visceral Adipose Tissue Is Associated With Recurrent Cardiovascular Events in Premature Coronary Artery Disease: Sub‐Analysis of the GEA Study Cohort.” *European Journal of Preventive Cardiology*, ahead of print, February 17. 10.1093/eurjpc/zwaf074. 39957360

[acel70278-bib-0046] Mogilenko, D. A. , O. Shpynov , P. S. Andhey , et al. 2021. “Comprehensive Profiling of an Aging Immune System Reveals Clonal GZMK+ CD8+ T Cells as Conserved Hallmark of Inflammaging.” Immunity 54: 99–115.e12. 10.1016/j.immuni.2020.11.005. 33271118

[acel70278-bib-0047] Morris, A. B. , M. W. Adelman , K. B. Bennion , et al. 2025. “Fgl2 Regulates FcγRIIB+CD8+ T Cell Responses During Infection.” JCI Insight 10: e186259. 10.1172/jci.insight.186259. 40197366 PMC11981615

[acel70278-bib-0048] Morris, A. B. , C. R. Farley , D. F. Pinelli , et al. 2020. “Signaling Through the Inhibitory Fc Receptor FcγRIIB Induces CD8+ T Cell Apoptosis to Limit T Cell Immunity.” Immunity 52: 136–150.e6. 10.1016/j.immuni.2019.12.006. 31940267 PMC7326381

[acel70278-bib-0049] Morris, A. B. , D. F. Pinelli , D. Liu , M. Wagener , and M. L. Ford . 2020. “Memory T Cell‐Mediated Rejection Is Mitigated by FcγRIIB Expression on CD8+ T Cells.” American Journal of Transplantation 20: 2206–2215. 10.1111/ajt.15837. 32154641 PMC7395896

[acel70278-bib-0050] Mukherjee, S. , M. E. C. Bruno , J. Oakes , et al. 2023. “Mechanisms of γδ T Cell Accumulation in Visceral Adipose Tissue With Aging.” Frontiers in Aging 4: 1258836. 10.3389/fragi.2023.1258836. 38274288 PMC10808514

[acel70278-bib-0051] Muñoz‐Rojas, A. R. , G. Wang , C. Benoist , and D. Mathis . 2024. “Adipose‐Tissue Regulatory T Cells Are a Consortium of Subtypes That Evolves With Age and Diet.” Proceedings of the National Academy of Sciences of the United States of America 121: e2320602121. 10.1073/pnas.2320602121. 38227656 PMC10823167

[acel70278-bib-0052] Nguyen, L. , and S. Shanmugan . 2024. “A Review Article: The Relationship Between Obesity and Colorectal Cancer.” Current Diabetes Reports 25: 8. 10.1007/s11892-024-01556-0. 39621160 PMC11611961

[acel70278-bib-0053] Nimmerjahn, F. , and J. V. Ravetch . 2008. “Fcgamma Receptors as Regulators of Immune Responses.” Nature Reviews Immunology 8: 34–47. 10.1038/nri2206. 18064051

[acel70278-bib-0054] Nishimura, S. , I. Manabe , M. Nagasaki , et al. 2009. “CD8+ Effector T Cells Contribute to Macrophage Recruitment and Adipose Tissue Inflammation in Obesity.” Nature Medicine 15: 914–920. 10.1038/nm.1964. 19633658

[acel70278-bib-0055] Olsen, O. E. , K. F. Wader , H. Hella , et al. 2015. “Activin A Inhibits BMP‐Signaling by Binding ACVR2A and ACVR2B.” Cell Communication and Signaling: CCS 13: 27. 10.1186/s12964-015-0104-z. 26047946 PMC4467681

[acel70278-bib-0056] Ou, M.‐Y. , H. Zhang , P.‐C. Tan , S.‐B. Zhou , and Q.‐F. Li . 2022. “Adipose Tissue Aging: Mechanisms and Therapeutic Implications.” Cell Death & Disease 13: 300. 10.1038/s41419-022-04752-6. 35379822 PMC8980023

[acel70278-bib-0057] Palmer, A. K. , M. Xu , Y. Zhu , et al. 2019. “Targeting Senescent Cells Alleviates Obesity‐Induced Metabolic Dysfunction.” Aging Cell 18: e12950. 10.1111/acel.12950. 30907060 PMC6516193

[acel70278-bib-0058] Pereira, B. I. , O. P. Devine , M. Vukmanovic‐Stejic , et al. 2019. “Senescent Cells Evade Immune Clearance via HLA‐E‐Mediated NK and CD8+ T Cell Inhibition.” Nature Communications 10: 2387. 10.1038/s41467-019-10335-5. PMC6547655 31160572

[acel70278-bib-0059] Pinjusic, K. , O. A. Dubey , O. Egorova , et al. 2022. “Activin‐A Impairs CD8 T Cell‐Mediated Immunity and Immune Checkpoint Therapy Response in Melanoma.” Journal for Immunotherapy of Cancer 10: e004533. 10.1136/jitc-2022-004533. 35580932 PMC9125758

[acel70278-bib-0060] Quinn, K. M. , A. Fox , K. L. Harland , et al. 2018. “Age‐Related Decline in Primary CD8+ T Cell Responses Is Associated With the Development of Senescence in Virtual Memory CD8+ T Cells.” Cell Reports 23: 3512–3524. 10.1016/j.celrep.2018.05.057. 29924995

[acel70278-bib-0061] Quinn, K. M. , T. Hussain , F. Kraus , et al. 2020. “Metabolic Characteristics of CD8+ T Cell Subsets in Young and Aged Individuals Are Not Predictive of Functionality.” Nature Communications 11: 2857. 10.1038/s41467-020-16633-7. PMC7275080 32504069

[acel70278-bib-0062] Raskov, H. , A. Orhan , J. P. Christensen , and I. Gögenur . 2021. “Cytotoxic CD8+ T Cells in Cancer and Cancer Immunotherapy.” British Journal of Cancer 124: 359–367. 10.1038/s41416-020-01048-4. 32929195 PMC7853123

[acel70278-bib-0063] Renkema, K. R. , G. Li , A. Wu , M. J. Smithey , and J. Nikolich‐Žugich . 2014. “Two Separate Defects Affecting True Naive or Virtual Memory T Cell Precursors Combine to Reduce Naive T Cell Responses With Aging.” Journal of Immunology 192: 151–159. 10.4049/jimmunol.1301453. PMC3925780 24293630

[acel70278-bib-0064] Reyes‐Farias, M. , J. Fos‐Domenech , D. Serra , L. Herrero , and D. Sánchez‐Infantes . 2021. “White Adipose Tissue Dysfunction in Obesity and Aging.” Biochemical Pharmacology 192: 114723. 10.1016/j.bcp.2021.114723. 34364887

[acel70278-bib-0065] Saadey, A. A. , A. Yousif , N. Osborne , et al. 2023. “Rebalancing TGFβ1/BMP Signals in Exhausted T Cells Unlocks Responsiveness to Immune Checkpoint Blockade Therapy.” Nature Immunology 24: 280–294. 10.1038/s41590-022-01384-y. 36543960

[acel70278-bib-0066] Sacks, H. , and M. E. Symonds . 2013. “Anatomical Locations of Human Brown Adipose Tissue.” Diabetes 62: 1783–1790. 10.2337/db12-1430. 23704519 PMC3661606

[acel70278-bib-0067] Santos, A. L. , and S. Sinha . 2021. “Obesity and Aging: Molecular Mechanisms and Therapeutic Approaches.” Ageing Research Reviews 67: 101268. 10.1016/j.arr.2021.101268. 33556548

[acel70278-bib-0068] Sárvári, A. K. , E. L. Van Hauwaert , L. K. Markussen , et al. 2021. “Plasticity of Epididymal Adipose Tissue in Response to Diet‐Induced Obesity at Single‐Nucleus Resolution.” Cell Metabolism 33: 437–453.e5. 10.1016/j.cmet.2020.12.004. 33378646

[acel70278-bib-0069] Schaum, N. , B. Lehallier , O. Hahn , et al. 2020. “Ageing Hallmarks Exhibit Organ‐Specific Temporal Signatures.” Nature 583: 596–602. 10.1038/s41586-020-2499-y. 32669715 PMC7757734

[acel70278-bib-0070] Shan, L. , L. L. van den Hoogen , J. Meeldijk , et al. 2020. “Increased Intra‐Articular Granzyme M May Trigger Local IFN‐λ1/IL‐29 Response in Rheumatoid Arthritis.” Clinical and Experimental Rheumatology 38: 220–226. 10.55563/clinexprheumatol/ffb107. 31172927

[acel70278-bib-0071] Smith, L. K. , G. M. Boukhaled , S. A. Condotta , et al. 2018. “Interleukin‐10 Directly Inhibits CD8+ T Cell Function by Enhancing N‐Glycan Branching to Decrease Antigen Sensitivity.” Immunity 48: 299–312.e5. 10.1016/j.immuni.2018.01.006. 29396160 PMC5935130

[acel70278-bib-0072] Smith, L. M. , P. T. Walsh , T. Rüdiger , et al. 2004. “EphA3 Is Induced by CD28 and IGF‐1 and Regulates Cell Adhesion.” Experimental Cell Research 292: 295–303. 10.1016/j.yexcr.2003.08.021. 14697337

[acel70278-bib-0073] So, J. , O. Strobel , J. Wann , et al. 2025. “Robust Single‐Nucleus RNA Sequencing Reveals Depot‐Specific Cell Population Dynamics in Adipose Tissue Remodeling During Obesity.” eLife 13: RP97981. 10.7554/eLife.97981. 39804687 PMC11729396

[acel70278-bib-0074] Srinivasan, S. , S. Mishra , K. K.‐H. Fan , et al. 2025. “Age‐Dependent bi‐Phasic Dynamics of Ly49+CD8+ Regulatory T Cell Population.” Aging Cell 24: e14461. 10.1111/acel.14461. 39696807 PMC11984669

[acel70278-bib-0075] Starbeck‐Miller, G. R. , V. P. Badovinac , D. L. Barber , and J. T. Harty . 2014. “Cutting Edge: Expression of FcγRIIB Tempers Memory CD8 T Cell Function In Vivo.” Journal of Immunology 192: 35–39. 10.4049/jimmunol.1302232. PMC3874719 24285839

[acel70278-bib-0076] Trapnell, C. , D. Cacchiarelli , J. Grimsby , et al. 2014. “The Dynamics and Regulators of Cell Fate Decisions Are Revealed by Pseudotemporal Ordering of Single Cells.” Nature Biotechnology 32: 381–386. 10.1038/nbt.2859. PMC4122333 24658644

[acel70278-bib-0077] Trim, W. , J. E. Turner , and D. Thompson . 2018. “Parallels in Immunometabolic Adipose Tissue Dysfunction With Ageing and Obesity.” Frontiers in Immunology 9: 169. 10.3389/fimmu.2018.00169. 29479350 PMC5811473

[acel70278-bib-0078] Vasamsetti, S. B. , N. Natarajan , S. Sadaf , J. Florentin , and P. Dutta . 2023. “Regulation of Cardiovascular Health and Disease by Visceral Adipose Tissue‐Derived Metabolic Hormones.” Journal of Physiology 601: 2099–2120. 10.1113/JP282728. 35661362 PMC9722993

[acel70278-bib-0079] Voskoboinik, I. , J. C. Whisstock , and J. A. Trapani . 2015. “Perforin and Granzymes: Function, Dysfunction and Human Pathology.” Nature Reviews. Immunology 15: 388–400. 10.1038/nri3839. 25998963

[acel70278-bib-0080] Wang, G. , G. Li , A. Song , et al. 2025. “Distinct Adipose Progenitor Cells Emerging With Age Drive Active Adipogenesis.” Science 388: eadj0430. 10.1126/science.adj0430. 40273250 PMC12445215

[acel70278-bib-0081] Weisberg, S. P. , D. McCann , M. Desai , M. Rosenbaum , R. L. Leibel , and A. W. Ferrante . 2003. “Obesity Is Associated With Macrophage Accumulation in Adipose Tissue.” Journal of Clinical Investigation 112: 1796–1808. 10.1172/JCI19246. 14679176 PMC296995

[acel70278-bib-0082] Winer, D. A. , S. Winer , L. Shen , et al. 2011. “B Cells Promote Insulin Resistance Through Modulation of T Cells and Production of Pathogenic IgG Antibodies.” Nature Medicine 17: 610–617. 10.1038/nm.2353. PMC3270885 21499269

[acel70278-bib-0083] Wolf, F. A. , P. Angerer , and F. J. Theis . 2018. “SCANPY: Large‐Scale Single‐Cell Gene Expression Data Analysis.” Genome Biology 19: 15. 10.1186/s13059-017-1382-0. 29409532 PMC5802054

[acel70278-bib-0084] Wu, X. , T. Li , R. Jiang , X. Yang , H. Guo , and R. Yang . 2023. “Targeting MHC‐I Molecules for Cancer: Function, Mechanism, and Therapeutic Prospects.” Molecular Cancer 22: 194. 10.1186/s12943-023-01899-4. 38041084 PMC10693139

[acel70278-bib-0085] Wu, Y. , Y. Sun , L. Chen , et al. 2024. “Dynamics of Single‐Nuclei Transcriptomic Profiling of Adipose Tissue From Diverse Anatomical Locations During Mouse Aging Process.” Scientific Reports 14: 16093. 10.1038/s41598-024-66918-w. 38997312 PMC11245496

[acel70278-bib-0086] Yu, G. , L.‐G. Wang , Y. Han , and Q.‐Y. He . 2012. “clusterProfiler: An R Package for Comparing Biological Themes Among Gene Clusters.” OMICS: A Journal of Integrative Biology 16: 284–287. 10.1089/omi.2011.0118. 22455463 PMC3339379

[acel70278-bib-0087] Yu, L. , Q. Wan , Q. Liu , et al. 2024. “IgG Is an Aging Factor That Drives Adipose Tissue Fibrosis and Metabolic Decline.” Cell Metabolism 36: 793–807.e5. 10.1016/j.cmet.2024.01.015. 38378001 PMC11070064

[acel70278-bib-0088] Zhang, W. , X. Su , S. Liu , et al. 2025. “Age‐Specific and Sex‐Specific Associations of Visceral Adipose Tissue With Metabolic Health Status and Cardiovascular Disease Risk.” Acta Diabetologica 62: 1261–1270. 10.1007/s00592-025-02447-w. 39792170

[acel70278-bib-0089] Zhang, Y. , Y. Jiang , X. Yang , Y. Huang , A. Pan , and Y. Liao . 2024. “Adipose Tissue Senescence: Biological Changes, Hallmarks and Therapeutic Approaches.” Mechanisms of Ageing and Development 222: 111988. 10.1016/j.mad.2024.111988. 39265709

[acel70278-bib-0090] Zheng, G. X. Y. , J. M. Terry , P. Belgrader , et al. 2017. “Massively Parallel Digital Transcriptional Profiling of Single Cells.” Nature Communications 8: 14049. 10.1038/ncomms14049. PMC5241818 28091601

[acel70278-bib-0091] Zheng, Y. , J. Zhao , Y. Shan , S. Guo , S. J. Schrodi , and D. He . 2023. “Role of the Granzyme Family in Rheumatoid Arthritis: Current Insights and Future Perspectives.” Frontiers in Immunology 14: 1137918. 10.3389/fimmu.2023.1137918. 36875082 PMC9977805

