# Supplementary material for: Single‐Cell RNA Sequencing Identifies Accumulation of Fcgr2b + Virtual Memory‐Like CD8 T Cells With Cytotoxic and Inflammatory Potential in Aged Mouse White Adipose Tissue
Source: Aging Cell. 2025 Oct 21;24(12):e70278. doi: 10.1111/acel.70278 (PMC12686556; doi:10.1111/acel.70278)
Supplement: Supplementary file 1 — Appendix S1: acel70278‐sup‐0001‐AppendixS1.pdf. [file ACEL-24-e70278-s001.pdf]

Supplementary Figures:

Supplementary Figure 1. Aging and HFD-induced obesity impair gWAT function. (a) Serum glucose levels during glucose tolerance test (GTT) and insulin tolerance test (ITT) over 120 minutes in male (a,c), and female (b,d) mice across different groups. (e) Fasting serum glucose levels. (f) Area under the curve (AUC) of serum glucose levels during ITT. (g) Serum adiponectin levels. Data are presented as mean ± SEM. Statistical significance was determined using two-way ANOVA followed by Tukey's HSD post hoc test. N = 5-8 mice per group. P-values < 0.05 were considered significant. $p < 0.05$ (*), $p < 0.01$ (**), $p < 0.001$ (***), $p < 0.0001$ (****).

Supplementary Figure 2. scRNA analysis of gWAT. Heatmap showing expression of top 10 differentially expressed genes across different cell types within SVF of gWAT.

Supplementary Figure 3. Cell-cell interactions within SVF. Dot plots representing incoming and outgoing signaling strengths between cell types within the SVF across different samples, inferred using Cellchat. Dot size reflects the number of cells within cell type. YM: ND-fed young male mice; YF: ND-fed young female mice; HFDM: HFD-fed young male mice; HFDF: HFD-fed young female mice; AM: ND-fed aged male mice; AF: ND-fed aged female mice.

Supplementary Figure 4. Differential incoming signaling to CD8 T cells. Dot plots representing top 100 differentially expressed L-R pairs showing incoming signaling to CD8 T cells from other cell types within the SVF of ND-fed young, HFD-fed young, and ND-fed aged mice, identified using MultiNicheNet. Dot color reflects the scaled expression of L-R pairs.

Supplementary Figure 5. Differentially expressed genes in CD8 T cells. Dot plot displaying upregulated genes in CD8 T cells within SVF of ND-fed aged mice (logFC > 1 and p-values ≤ 0.05). Dot size represents the percentage of CD8 T cells expressing each gene and color indicates the average expression level.

Supplementary Figure 6. scRNA analysis of CD8 T cells. Heatmap showing expression of the top 10 differentially expressed genes across CD8 T cell subsets.

Supplementary Figure 7. Differential outgoing signaling from CD8 T cells. Dot plots representing top 50 differentially expressed L-R pairs showing outgoing signaling from CD8 T

cells to other cell types within the SVF of ND-fed young, HFD-fed young, and ND-fed aged mice, inferred using MultiNicheNet. Dot color reflects the scaled expression of L-R pairs.

Supplementary Figure 8. Gating strategy used to identify CD8 T cell subsets using flow cytometry.

Supplementary Figure 9. (a) Histograms representing fluorescent intensities of SA-β-gal in cycling and senescent cells detected using flow cytometry. (b) Bar graphs representing frequencies of SA-β-gal positive cells in cycling and senescent cells. Data are presented as mean ± SEM. Statistical significance was determined using unpaired t-test. p-values < 0.05 were considered significant. $p < 0.0001$ (****).

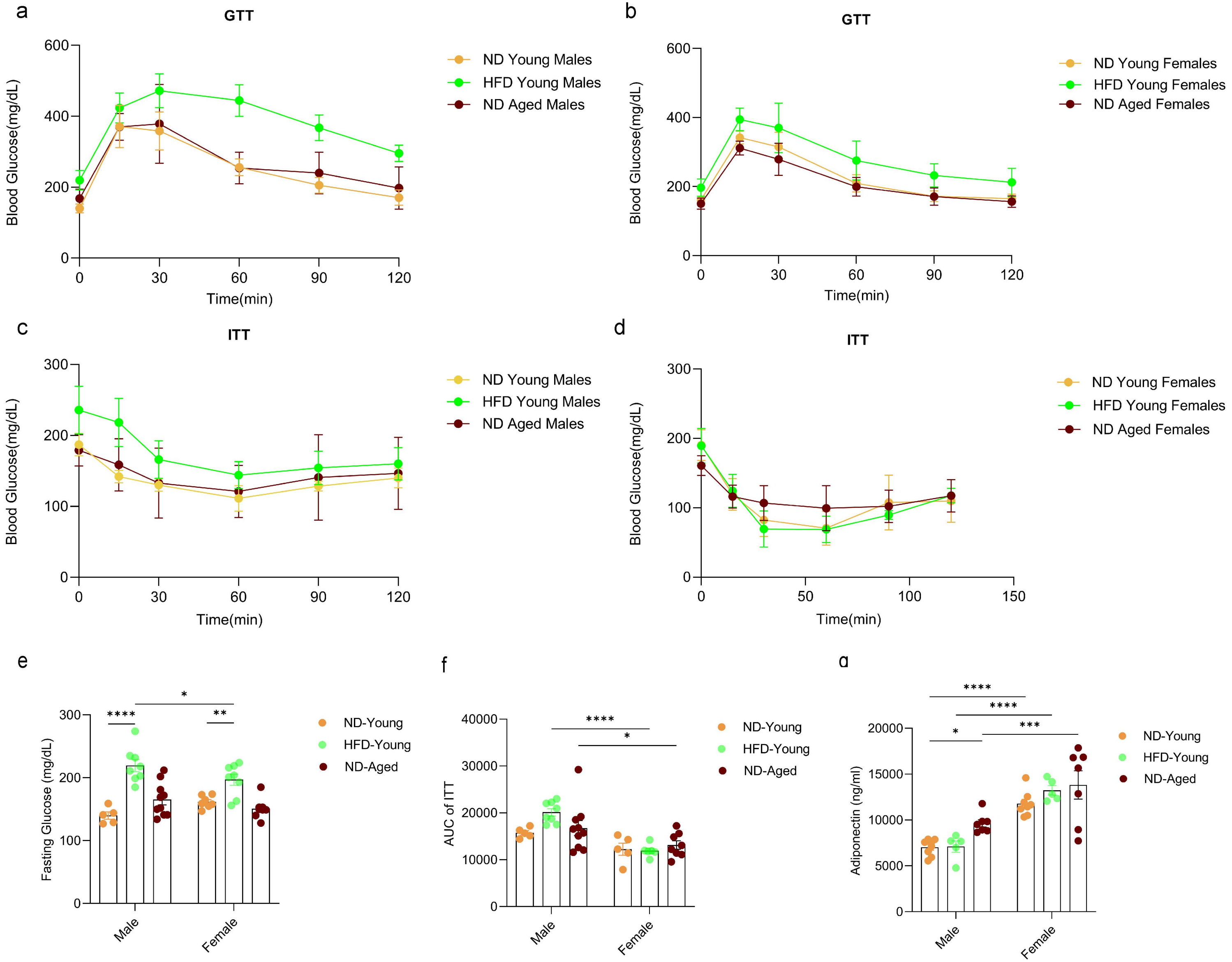
a
GTT
Blood Glucose(mg/dL)
Time(min)
ND Young Males
HFD Young Males
ND Aged Males
b
GTT
Blood Glucose(mg/dL)
Time(min)
ND Young Females
HFD Young Females
ND Aged Females
c
ITT
Blood Glucose(mg/dL)
Time(min)
ND Young Males
HFD Young Males
ND Aged Males
d
ITT
Blood Glucose(mg/dL)
Time(min)
ND Young Females
HFD Young Females
ND Aged Females
e
Fasting Glucose (mg/dL)
Male
Female
ND-Young
HFD-Young
ND-Aged
f
AUC of ITT
Male
Female
ND-Young
HFD-Young
ND-Aged
g
Adiponectin (ng/ml)
Male
Female
ND-Young
HFD-Young
ND-Aged

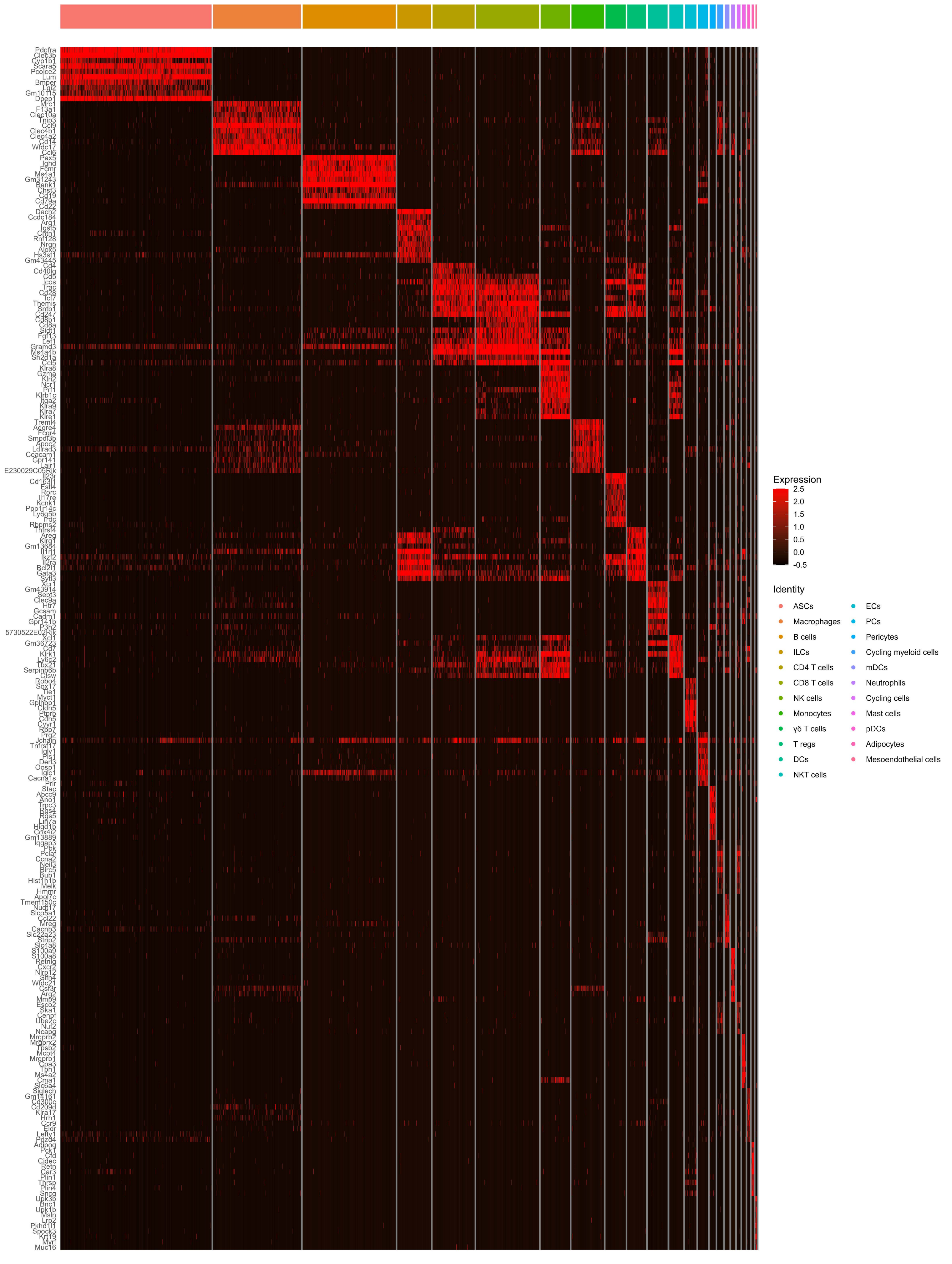

Pdgfra
Clec3b
Cyp1b1
Scara5
Pcolce2
Lum
Bmper
Lgi2
Gm10115
Dpep1
Mrc1
F13a1
Clec10a
Tnip3
Ccl9
Clec4b1
Clec4a2
Cd14
Wfdc17
Ccl6
Pax5
Ighd
Fcmr
Ms4a1
Gm31243
Bank1
Chst3
Cd19
Cd79a
Cd22
Dach2
Ccdc184
Arg1
Igsf5
Cntn1
Rnf128
Nrgn
Alox5
Hs3st1
Gm43445
Cd4
Cd40lg
Cd5
Icos
Trac
Cd28
Tcf7
Themis
Sntb1
Cd247
Cd8b1
Cd8a
Sidt1
Fgf13
Lef1
Gramd3
Ms4a4b
Sh2d1a
Ccl5
Klra8
Gzma
Klri2
Ncr1
Prf1
Klrb1c
Itga2
Klra9
Klra7
Klre1
Treml4
Adgre4
Fcgr4
Smpdl3b
Apoc2
Ldlrad3
Ceacam1
Gpr141
Lair1
E230029C05Rik
Il23r
Cd163l1
Fstl4
Rorc
Il17re
Kcnk1
Ppp1r14c
Ly6g5b
Trdc
Rbpms2
Tnfrsf4
Areg
Klrg1
Gm13684
Il1rl1
Ikzf2
Il2ra
Bcl2l1
Gata3
Sytl3
Xcr1
Gm43914
Sept3
Clec9a
Htr7
Gcsam
Cadm1
Gpr141b
P3h2
5730522E02Rik
Xcl1
Gm36723
Cd7
Klrk1
Ly6c2
Tbx21
Serpinb6b
Ctsw
Robo4
Sox17
Tie1
Myct1
Gpihbp1
Cldn5
Ptprb
Cdh5
Cyyr1
Rbp7
Prg2
Jchain
Tnfrsf17
Iglv1
Pls1
Derl3
Oosp1
Iglc1
Cacna1s
Prlr
Stac
Abcc9
Ano1
Trpc3
Rgs4
Rgs5
Lin7a
Higd1b
Cox4i2
Gm13889
Iqgap3
Pbk
Pclaf
Ccna2
Neil3
Birc5
Bub1
Hist1h1b
Melk
Hmmr
Apol7c
Tmem150c
Nudt17
Slco5a1
Ccl22
Mreg
Cacnb3
Slc22a23
Strip2
Slc4a8
S100a9
S100a8
Retnlg
Cxcr2
Nlrp12
Slfn4
Wfdc21
Csf3r
Arg2
Mmp9
Esco2
Ska1
Cenpf
Ube2c
Nuf2
Ncapg
Mrgprb2
Mrgprx2
Tpsb2
Mcpt4
Mrgprb1
Cpa3
Tph1
Ms4a2
Cma1
Slc6a4
Siglech
Gm14161
Cd300c
Cd209d
Klra17
Hrh1
Ccr9
Eldr
Lefty1
Pdzd4
Adipoq
Pck1
Cfd
Cidec
Retn
Car3
Plin1
Thrsp
Plin4
Sncg
Upk3b
Bnc1
Upk1b
Msln
Lrp2
Pkhd1l1
Spock3
Krt19
Myrf
Muc16
Expression
2.5
2.0
1.5
1.0
0.5
0.0
-0.5
Identity
ASCs
Macrophages
B cells
ILCs
CD4 T cells
CD8 T cells
NK cells
Monocytes
γδ T cells
T regs
DCs
NKT cells
ECs
PCs
Pericytes
Cycling myeloid cells
mDCs
Neutrophils
Cycling cells
Mast cells
pDCs
Adipocytes
Mesoendothelial cells

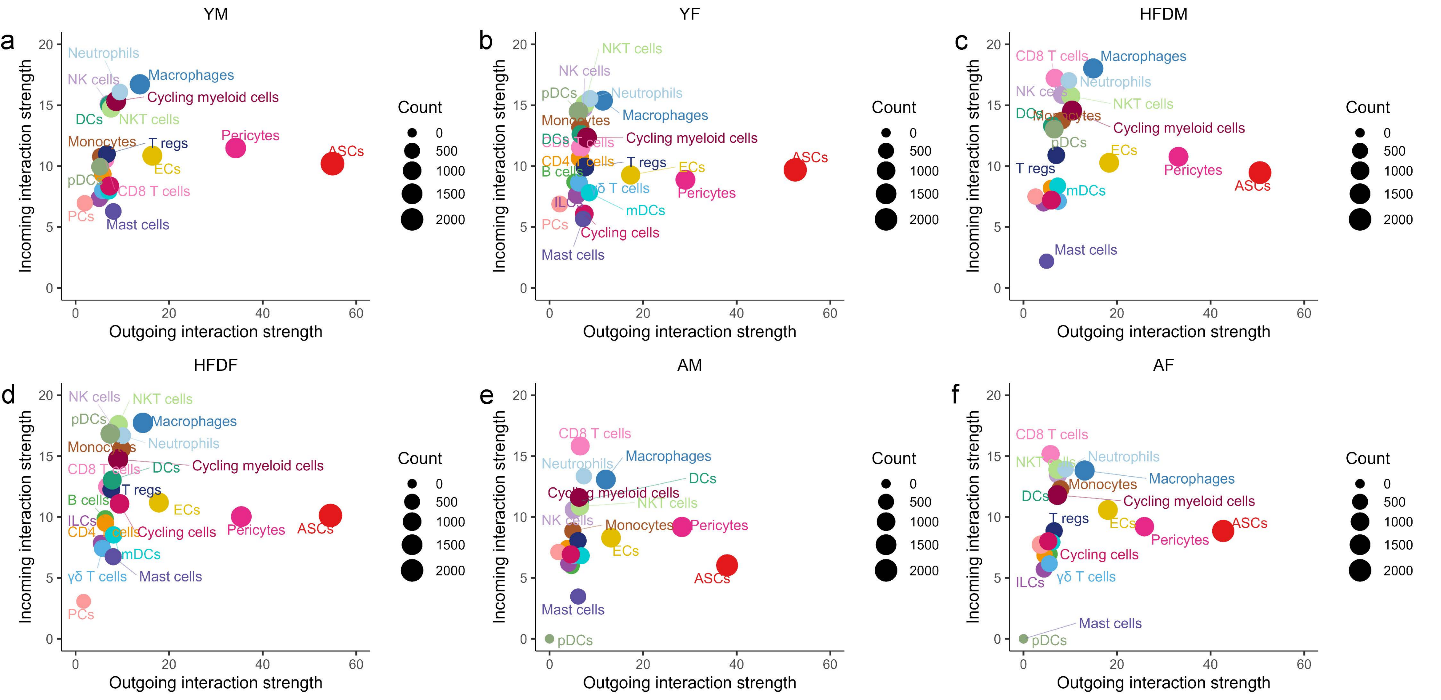
YM
YF
HFDM
HFDF
AM
AF
a
b
c
d
e
f
Incoming interaction strength
Outgoing interaction strength
0
5
10
15
20
0
20
40
60
Count
0
500
1000
1500
2000
Neutrophils
NK cells
Macrophages
Cycling myeloid cells
DCs
NKT cells
Monocytes
T regs
Pericytes
ASCs
ECs
pDCs
CD8 T cells
PCs
Mast cells
CD4 T cells
B cells
γδ T cells
mDCs
ILCs
Cycling cells

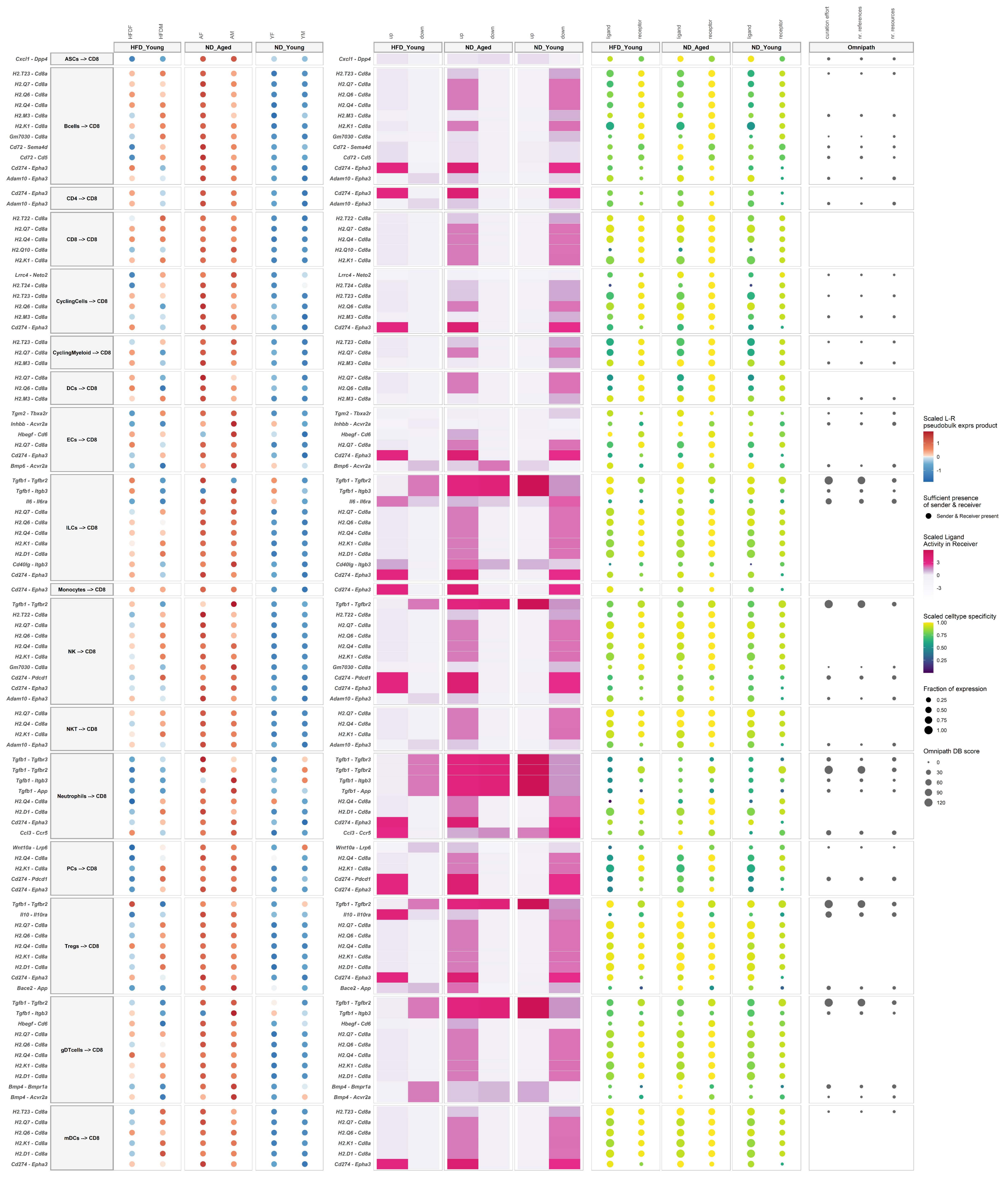

HFDF
HFDM
AF
AM
YF
YM
HFD_Young
ND_Aged
ND_Young
up
down
ligand
receptor
curation effort
nr. references
nr. resources
Omnipath
Cxcl1 - Dpp4
ASCs --> CD8
H2.T23 - Cd8a
H2.Q7 - Cd8a
H2.Q6 - Cd8a
H2.Q4 - Cd8a
H2.M3 - Cd8a
H2.K1 - Cd8a
Gm7030 - Cd8a
Cd72 - Sema4d
Cd72 - Cd5
Cd274 - Epha3
Adam10 - Epha3
Bcells --> CD8
Cd274 - Epha3
Adam10 - Epha3
CD4 --> CD8
H2.T22 - Cd8a
H2.Q7 - Cd8a
H2.Q4 - Cd8a
H2.Q10 - Cd8a
H2.K1 - Cd8a
CD8 --> CD8
Lrrc4 - Neto2
H2.T24 - Cd8a
H2.T23 - Cd8a
H2.Q6 - Cd8a
H2.M3 - Cd8a
Cd274 - Epha3
CyclingCells --> CD8
H2.T23 - Cd8a
H2.Q7 - Cd8a
H2.M3 - Cd8a
CyclingMyeloid --> CD8
H2.Q7 - Cd8a
H2.Q6 - Cd8a
H2.M3 - Cd8a
DCs --> CD8
Tgm2 - Tbxa2r
Inhbb - Acvr2a
Hbegf - Cd6
H2.Q7 - Cd8a
Cd274 - Epha3
Bmp6 - Acvr2a
ECs --> CD8
Tgfb1 - Tgfbr2
Tgfb1 - Itgb3
Il6 - Il6ra
H2.Q7 - Cd8a
H2.Q6 - Cd8a
H2.Q4 - Cd8a
H2.K1 - Cd8a
H2.D1 - Cd8a
Cd40lg - Itgb3
Cd274 - Epha3
ILCs --> CD8
Cd274 - Epha3
Monocytes --> CD8
Tgfb1 - Tgfbr2
H2.T22 - Cd8a
H2.Q7 - Cd8a
H2.Q6 - Cd8a
H2.Q4 - Cd8a
H2.K1 - Cd8a
Gm7030 - Cd8a
Cd274 - Pdcd1
Cd274 - Epha3
Adam10 - Epha3
NK --> CD8
H2.Q7 - Cd8a
H2.Q4 - Cd8a
H2.K1 - Cd8a
Adam10 - Epha3
NKT --> CD8
Tgfb1 - Tgfbr3
Tgfb1 - Tgfbr2
Tgfb1 - Itgb3
Tgfb1 - App
H2.Q4 - Cd8a
H2.D1 - Cd8a
Cd274 - Epha3
Ccl3 - Ccr5
Neutrophils --> CD8
Wnt10a - Lrp6
H2.Q4 - Cd8a
H2.K1 - Cd8a
Cd274 - Pdcd1
Cd274 - Epha3
PCs --> CD8
Tgfb1 - Tgfbr2
Il10 - Il10ra
H2.Q7 - Cd8a
H2.Q6 - Cd8a
H2.Q4 - Cd8a
H2.K1 - Cd8a
H2.D1 - Cd8a
Cd274 - Epha3
Bace2 - App
Tregs --> CD8
Tgfb1 - Tgfbr2
Tgfb1 - Itgb3
Hbegf - Cd6
H2.Q7 - Cd8a
H2.Q6 - Cd8a
H2.Q4 - Cd8a
H2.K1 - Cd8a
H2.D1 - Cd8a
Bmp4 - Bmpr1a
Bmp4 - Acvr2a
gDTcells --> CD8
H2.T23 - Cd8a
H2.Q7 - Cd8a
H2.Q6 - Cd8a
H2.K1 - Cd8a
H2.D1 - Cd8a
Cd274 - Epha3
mDCs --> CD8
Scaled L-R pseudobulk exprs product
1
0
-1
Sufficient presence of sender & receiver
Sender & Receiver present
Scaled Ligand Activity in Receiver
3
0
-3
Scaled celltype specificity
1.00
0.75
0.50
0.25
Fraction of expression
0.25
0.50
0.75
1.00
Omnipath DB score
0
30
60
90
120

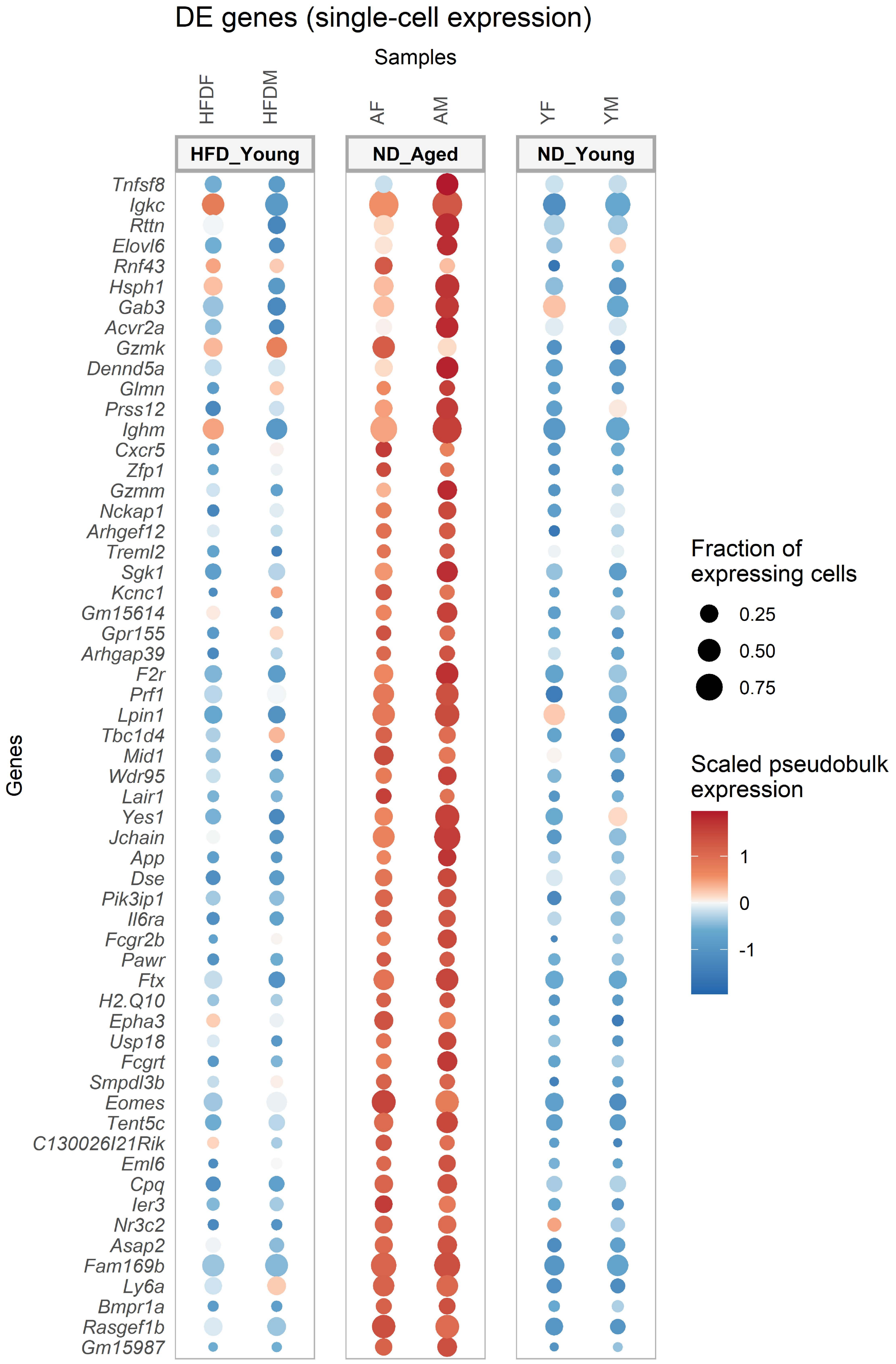
DE genes (single-cell expression)
Samples
HFDF
HFDM
AF
AM
YF
YM
HFD_Young
ND_Aged
ND_Young
Genes
Tnfsf8
Igkc
Rttn
Elovl6
Rnf43
Hsph1
Gab3
Acvr2a
Gzmk
Dennd5a
Glmn
Prss12
Ighm
Cxcr5
Zfp1
Gzmm
Nckap1
Arhgef12
Treml2
Sgk1
Kcnc1
Gm15614
Gpr155
Arhgap39
F2r
Prf1
Lpin1
Tbc1d4
Mid1
Wdr95
Lair1
Yes1
Jchain
App
Dse
Pik3ip1
Il6ra
Fcgr2b
Pawr
Ftx
H2.Q10
Epha3
Usp18
Fcgrt
Smpdl3b
Eomes
Tent5c
C130026I21Rik
Eml6
Cpq
Ier3
Nr3c2
Asap2
Fam169b
Ly6a
Bmpr1a
Rasgef1b
Gm15987
Fraction of expressing cells
0.25
0.50
0.75
Scaled pseudobulk expression
1
0
-1

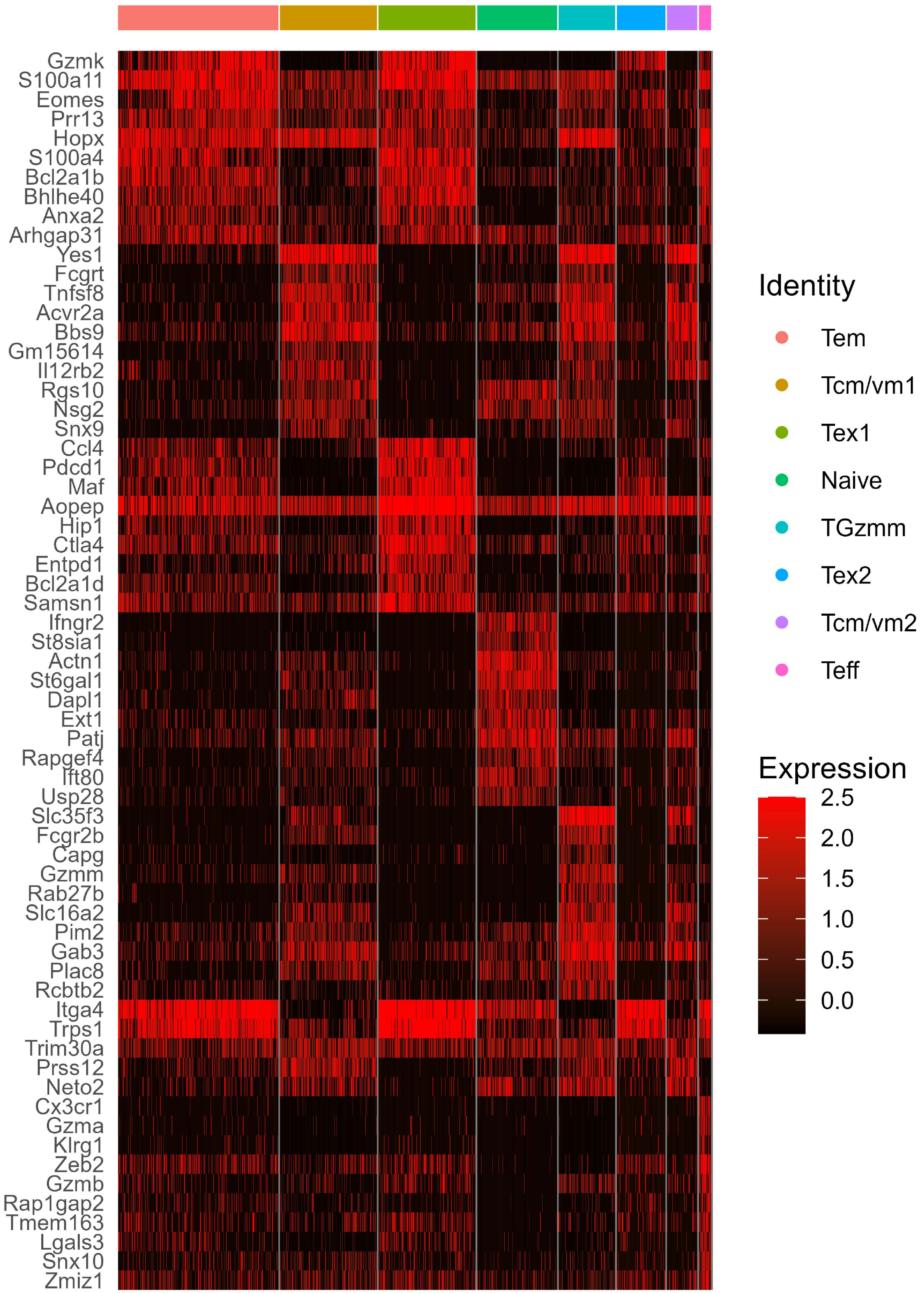
Gzmk
S100a11
Eomes
Prr13
Hopx
S100a4
Bcl2a1b
Bhlhe40
Anxa2
Arhgap31
Yes1
Fcgrt
Tnfsf8
Acvr2a
Bbs9
Gm15614
Il12rb2
Rgs10
Nsg2
Snx9
Ccl4
Pdcd1
Maf
Aopep
Hip1
Ctla4
Entpd1
Bcl2a1d
Samsn1
Ifngr2
St8sia1
Actn1
St6gal1
Dapl1
Ext1
Patj
Rapgef4
Ift80
Usp28
Slc35f3
Fcgr2b
Capg
Gzmm
Rab27b
Slc16a2
Pim2
Gab3
Plac8
Rcbtb2
Itga4
Trps1
Trim30a
Prss12
Neto2
Cx3cr1
Gzma
Klrg1
Zeb2
Gzmb
Rap1gap2
Tmem163
Lgals3
Snx10
Zmiz1
Identity
Tem
Tcm/vm1
Tex1
Naive
TGzmm
Tex2
Tcm/vm2
Teff
Expression
2.5
2.0
1.5
1.0
0.5
0.0

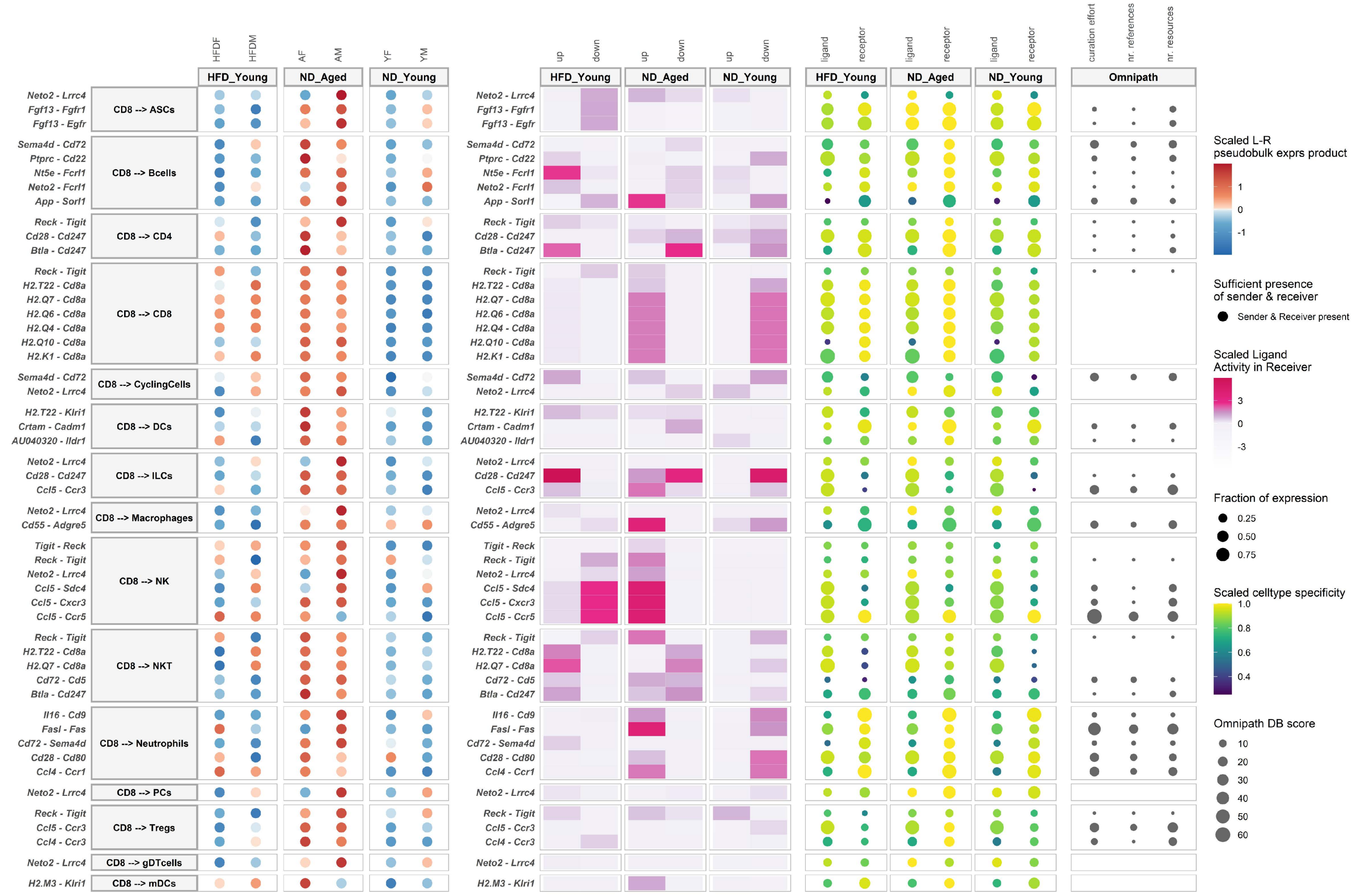

HFDF
HFDM
AF
AM
YF
YM
HFD_Young
ND_Aged
ND_Young
up
down
ligand
receptor
curation effort
nr. references
nr. resources
Omnipath
Neto2 - Lrrc4
Fgf13 - Fgfr1
Fgf13 - Egfr
CD8 --> ASCs
Sema4d - Cd72
Ptprc - Cd22
Nt5e - Fcrl1
Neto2 - Fcrl1
App - Sorl1
CD8 --> Bcells
Reck - Tigit
Cd28 - Cd247
Btla - Cd247
CD8 --> CD4
Reck - Tigit
H2.T22 - Cd8a
H2.Q7 - Cd8a
H2.Q6 - Cd8a
H2.Q4 - Cd8a
H2.Q10 - Cd8a
H2.K1 - Cd8a
CD8 --> CD8
Sema4d - Cd72
Neto2 - Lrrc4
CD8 --> CyclingCells
H2.T22 - Klri1
Crtam - Cadm1
AU040320 - Ildr1
CD8 --> DCs
Neto2 - Lrrc4
Cd28 - Cd247
Ccl5 - Ccr3
CD8 --> ILCs
Neto2 - Lrrc4
Cd55 - Adgre5
CD8 --> Macrophages
Tigit - Reck
Reck - Tigit
Neto2 - Lrrc4
Ccl5 - Sdc4
Ccl5 - Cxcr3
Ccl5 - Ccr5
CD8 --> NK
Reck - Tigit
H2.T22 - Cd8a
H2.Q7 - Cd8a
Cd72 - Cd5
Btla - Cd247
CD8 --> NKT
Il16 - Cd9
Fasl - Fas
Cd72 - Sema4d
Cd28 - Cd80
Ccl4 - Ccr1
CD8 --> Neutrophils
Neto2 - Lrrc4
CD8 --> PCs
Reck - Tigit
Ccl5 - Ccr3
Ccl4 - Ccr3
CD8 --> Tregs
Neto2 - Lrrc4
CD8 --> gDTcells
H2.M3 - Klri1
CD8 --> mDCs
Scaled L-R pseudobulk exprs product
1
0
-1
Sufficient presence of sender & receiver
Sender & Receiver present
Scaled Ligand Activity in Receiver
3
0
-3
Fraction of expression
0.25
0.50
0.75
Scaled celltype specificity
1.0
0.8
0.6
0.4
Omnipath DB score
10
20
30
40
50
60

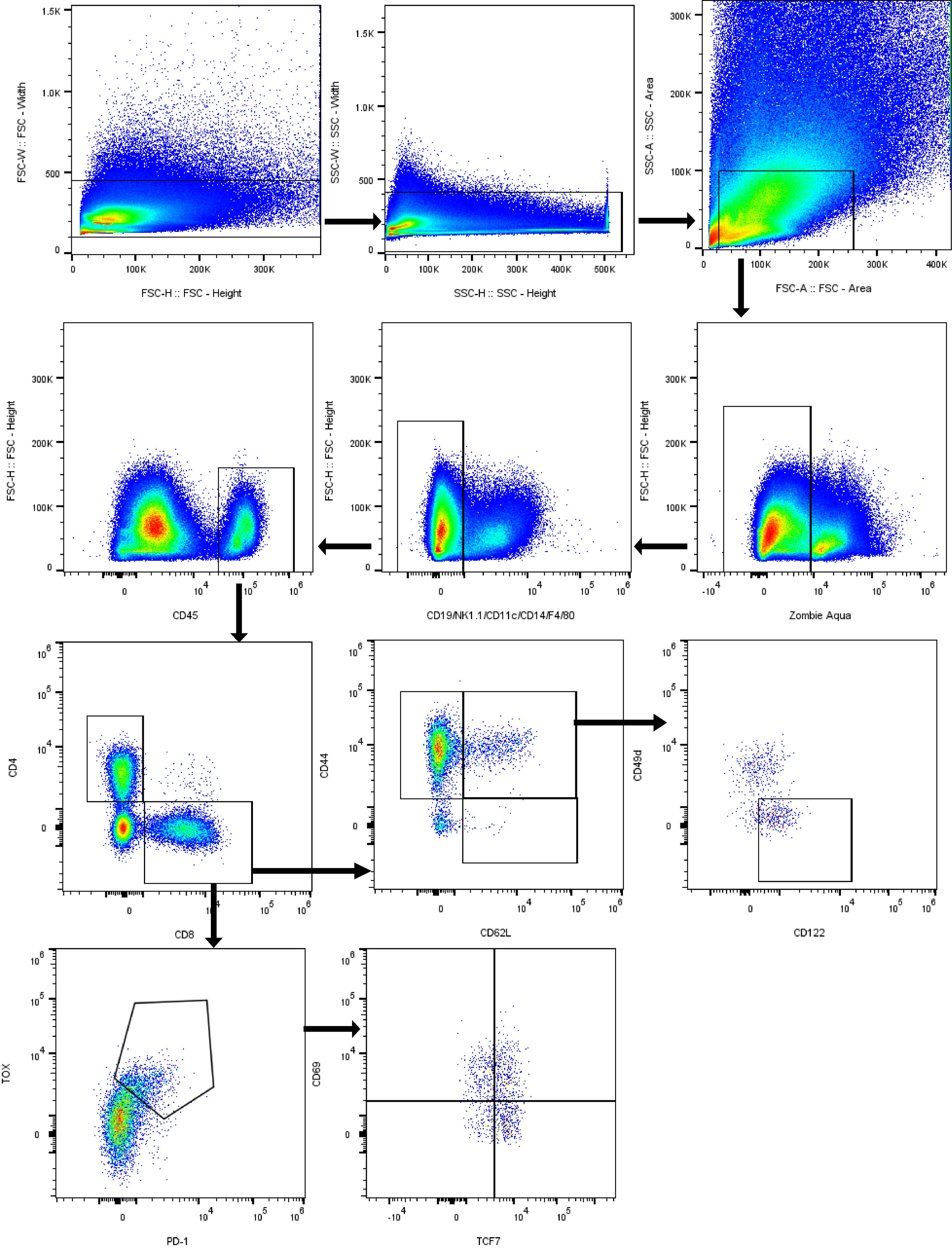
FSC-W :: FSC - Width
FSC-H :: FSC - Height
SSC-W :: SSC - Width
SSC-H :: SSC - Height
SSC-A :: SSC - Area
FSC-A :: FSC - Area
FSC-H :: FSC - Height
Zombie Aqua
CD19/NK1.1/CD11c/CD14/F4/80
CD45
CD4
CD8
CD44
CD62L
CD49d
CD122
TOX
PD-1
CD69
TCF7

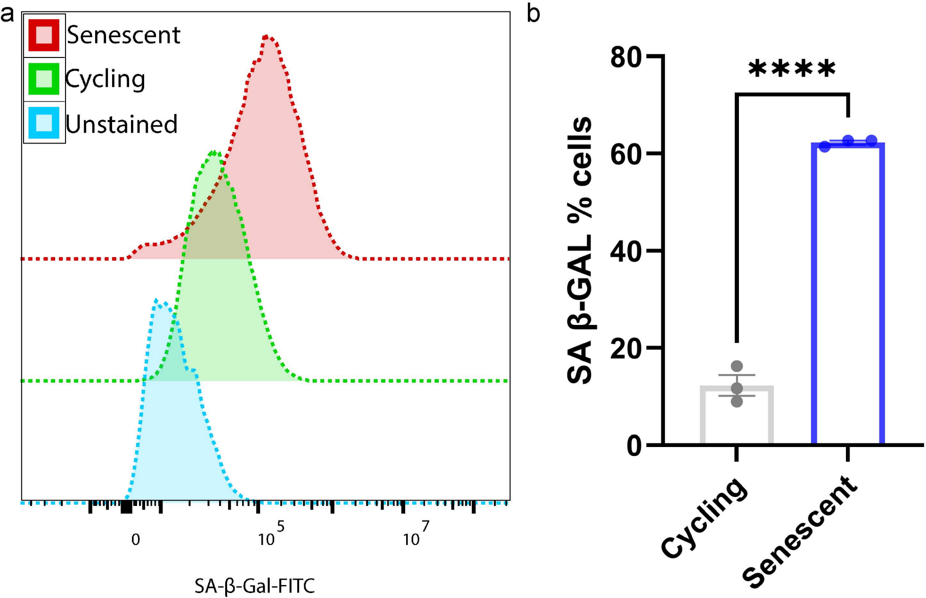

a
Senescent
Cycling
Unstained
0
10^5
10^7
SA-β-Gal-FITC
b
SA β-GAL % cells
80
60
40
20
0
****
Cycling
Senescent
